# Heterozygous *COL17A1* variants are a frequent cause of amelogenesis imperfecta

**DOI:** 10.1136/jmg-2023-109510

**Published:** 2023-11-18

**Authors:** Ummey Hany, Christopher M Watson, Lu Liu, Claire E L Smith, Asmaa Harfoush, James A Poulter, Georgios Nikolopoulos, Richard Balmer, Catriona J Brown, Anesha Patel, Jenny Simmonds, Ruth Charlton, María Gabriela Acosta de Camargo, Helen D Rodd, Hussain Jafri, Agne Antanaviciute, Michelle Moffat, Maisoon Al-Jawad, Chris F Inglehearn, Alan J Mighell

**Affiliations:** 1 Leeds Institute of Medical Research, University of Leeds, St. James's University Hospital, Leeds, UK; 2 North East and Yorkshire Genomic Laboratory Hub, Central Lab, St. James's University Hospital, Leeds, UK; 3 School of Dentistry, Clarendon Way, University of Leeds, Leeds, UK; 4 Institute for Fundamental Biomedical Research, B.S.R.C. 'Alexander Fleming', Vari, Attica, Greece; 5 Birmingham Dental Hospital, Mill Pool Way, Edgbaston, Birmingham, UK; 6 LCRN West Midlands Core Team, NIHR Clinical Research Network (CRN), Birmingham Research Park (West Wing), Vincent Drive, Edgbaston, Birmingham, UK; 7 Department of Dentistry of the Child and Adolescent, Universidad de Carabobo, Carabodo, Venezuela; 8 Academic Unit of Oral Health Dentistry and Society, School of Clinical Dentistry, University of Sheffield, Sheffield, UK; 9 Fatima Jinnah Medical University, Punjab Thalassaemia and Other Genetic Disorders Prevention and Research Institute, Lahore, Pakistan; 10 MRC Human Immunology Unit, University of Oxford, Oxford, UK; 11 Paediatric Dentistry, The Newcastle Upon Tyne Hospitals NHS Foundation Trust, Newcastle upon Tyne, UK

**Keywords:** Collagen XVII, COL17A1, amelogenesis imperfecta, junctional epidermolysis bullosa

## Abstract

**Background:**

Collagen XVII is most typically associated with human disease when biallelic *COL17A1* variants (>230) cause junctional epidermolysis bullosa (JEB), a rare, genetically heterogeneous, mucocutaneous blistering disease with amelogenesis imperfecta (AI), a developmental enamel defect. Despite recognition that heterozygous carriers in JEB families can have AI, and that heterozygous *COL17A1* variants also cause dominant corneal epithelial recurrent erosion dystrophy (ERED), the importance of heterozygous *COL17A1* variants causing dominant non-syndromic AI is not widely recognised.

**Methods:**

Probands from an AI cohort were screened by single molecule molecular inversion probes or targeted hybridisation capture (both a custom panel and whole exome sequencing) for *COL17A1* variants. Patient phenotypes were assessed by clinical examination and analyses of affected teeth.

**Results:**

Nineteen unrelated probands with isolated AI (no co-segregating features) had 17 heterozygous, potentially pathogenic *COL17A1* variants, including missense, premature termination codons, frameshift and splice site variants in both the endo-domains and the ecto-domains of the protein. The AI phenotype was consistent with enamel of near normal thickness and variable focal hypoplasia with surface irregularities including pitting.

**Conclusion:**

These results indicate that *COL17A1* variants are a frequent cause of dominantly inherited non-syndromic AI. Comparison of variants implicated in AI and JEB identifies similarities in type and distribution, with five identified in both conditions, one of which may also cause ERED. Increased availability of genetic testing means that more individuals will receive reports of heterozygous *COL17A1* variants. We propose that patients with isolated AI or ERED, due to *COL17A1* variants, should be considered as potential carriers for JEB and counselled accordingly, reflecting the importance of multidisciplinary care.

**WHAT IS ALREADY KNOWN ON THIS TOPIC**

- There is an established understanding that biallelic *COL17A1* variants are a cause of junctional epidermolysis bullosa (JEB).

**WHAT THIS STUDY ADDS**

- Heterozygous *COL17A1* variants are a much more common cause than previously recognised of isolated autosomal dominant amelogenesis imperfecta (AI) (developmental enamel defects) in the absence of mucocutaneous disease.

**HOW THIS STUDY MIGHT AFFECT RESEARCH, PRACTICE OR POLICY**

- As genetic testing availability increases, including as part of AI and corneal dystrophy care, more individuals will receive reports of heterozygous *COL17A1* variants.
- This study provides a reference point to inform how genetic counselling and clinical care are advanced.
- Furthermore, there is a need to consider specialist dental and ophthalmic evaluation of carriers in JEB families to ensure that their care needs are also being met.

## Introduction

Collagen type XVII alpha 1 chain (COL17A1), hereafter referred to as collagen XVII, is a hemidesmosomal transmembrane protein widely expressed in humans. It has diverse biological functions in cell adhesion, morphogenesis, neuromuscular signalling and host defence.^1^ As a hemidesmosome component, it is present in the cutaneous basement membrane zone, which connects the skin epidermis and dermis.^2 3^ It is also expressed during amelogenesis, the process by which dental enamel is formed, and contributes to the differentiation of ameloblasts.^4 5^ Col17 knockout (Col17^−/−^) mice exhibit distorted Tomes’ processes, a reduced volume of enamel matrix during the secretory stage and prolonged calcification in the maturation stage of amelogenesis.^4^

**Figure 1:**
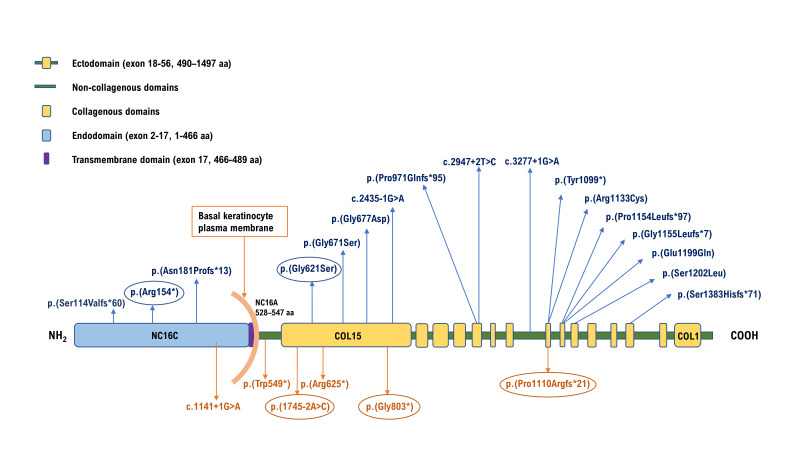
Schematic representation of the domain organisation of the collagen XVII protein. The extracellular domain or ectodomain is comprised of 15 collagenous (COL1–COL15, yellow vertical boxes) flanked by stretches of non-collagen sequence (NC1–NC16a, green horizontal lines). The non-collagen domain, NC16, spans from the extracellular matrix to cytoplasm and comprises a transmembrane domain NC16b adjoined by NC16a and NC16c to the C-terminal and N-terminal ends respectively. *COL17A1* variants identified in this study are denoted in blue text above the protein domains and variants published by others as causes of amelogenesis imperfecta are displayed in orange text below the protein domains.^25 50^ The circled variants have been previously published in association with junctional epidermolysis bullosa.

The 56 exons of the *COL17A1* gene encode a 1497-amino acid protein which acts as a homotrimer composed of three alpha (α1) chains, each with a molecular mass of 180 kDa. These consist of a globular amino-terminal intracellular endodomain, a short transmembrane domain and a flexible rod-like carboxy-terminal extracellular ectodomain.^6^ The ectodomain consists of 15 collagenous (COL1–COL15) sequences containing repeating Gly-X-Y tripeptides which, in the homotrimer, form the characteristic collagen triple helices. These are flanked by 16 non-collagenous sequences (NC1–NC16) (figure 1).^7^ A notable characteristic of collagen XVII is the shedding of the ectodomain after cleavage at the cell surface by the sheddases ADAM 9, 10 and 17, to yield its soluble intracellular form; the biological significance of this remains to be determined.^8^

Biallelic variants in *COL17A1* (OMIM 113811) are a well-documented cause of the recessively inherited, genetically heterogeneous mucocutaneous blistering condition junctional epidermolysis bullosa (JEB).^9^ JEB is genetically heterogeneous and characterised by erosions and blistering of the skin and mucous membranes, with cleavage at the basement membrane zone. There are a range of clinical presentations, but it is generally classified into one of two major subtypes: intermediate or severe.^10^ JEB prevalence is estimated to be approximately 2 per million live births in the USA and 1 per million in England and Wales.^11–13^

Corneal epithelial erosions and enamel hypoplasia are distinct features in JEB families.^13^ Corneal erosions are found in only a proportion of JEB cases,^14^ while it has been reported that enamel hypoplasia is always associated with JEB.^15^ Hintner and Wolff^16^ first reported defective enamel in their patients with JEB, and since then, enamel hypoplasia in association with JEB has been further corroborated.^17 18^ In comparison to healthy enamel, the enamel of patients with JEB has increased tissue porosity, reduced mineral content and contains serum albumin, with enamel hypoplasia.^19^ These studies identified JEB on clinical features, without knowing which of the genes known to cause JEB was responsible. In papers primarily about JEB due to biallelic *COL17A1* changes, carrier parents or siblings of patients with JEB have been described as having developmental enamel defects, typically using the non-specific descriptive term enamel hypoplasia.^20 21^

Monoallelic *COL17A1* variants can also cause dominantly inherited epithelial recurrent erosion dystrophy (ERED, OMIM 122400), a corneal disease with the potential for lifelong progression and vision loss (three variants reported).^22 23^ Furthermore, there are documented cases of monoallelic *COL17A1* variants causing dominantly inherited amelogenesis imperfecta (AI) in the absence of other co-segregating features or any family history of JEB. AI is a developmental failure of normal dental enamel formation affecting all teeth, which can be inherited as a dominant, recessive or X-linked trait, either in isolation or as a component of syndromic conditions.^24^ Dominant isolated AI caused by heterozygous *COL17A1* variants has only been reported in two cohort studies, where *COL17A1* was only one of several genes implicated, and in one report of a genetically complex AI family (total six variants),^25–27^ and *COL17A1* variants were not listed as a cause of AI in OMIM at the time of submission (13 July 2023). The distinction between AI and descriptive terms such as enamel hypoplasia is important. The latter term does not link to aetiology or inheritance, unlike AI.

Here, we describe 19 unrelated families with isolated AI in which probands are heterozygous for 17 different monoallelic *COL17A1* variants, consistent with this being a frequent cause of autosomal dominant AI presenting in the absence of other clinical features.

## Materials and methods

### Patient recruitment

Patients were recruited though UK dental clinics, with informed written consent and local ethical approval (REC 13/YH/0028), in accordance with the principles of the Declaration of Helsinki. Genomic DNA was obtained from venous blood using conventional extraction techniques, or from saliva using Oragene DNA Sample Collection kits (DNA Genotek). Screening of families F2-F19 was carried out by the University of Leeds Amelogenesis Research Group, while family F1 was screened via the NHS testing service for AI (https://www.england.nhs.uk/publication/national-genomic-test-directories/).

10.1136/jmg-2023-109510.supp2 Supplementary data

### Reference genome and transcript

The human reference genome used for this study was GRCh37/hg19, the transcript sequence of *COL17A1* used was NM_000494.4 and the collagen XVII protein sequence used was NP_000485.

### Whole exome sequencing (WES)

Two different hybridisation capture reagents were used for WES library preparation: the SureSelect Human All Exon V6 kit (Agilent Technologies) and the Human Comprehensive Exome kit (10–50 Mb) (Twist Bioscience). For SureSelect, 3 µg genomic DNA was used to make libraries, following the manufacturer’s protocol. These were sequenced on a HiSeq 3000 which generated paired-end 150 bp reads (Illumina). For the Twist kit, 50 ng genomic DNA was used to make libraries following the manufacturer’s protocol. Sequencing was carried out on a NextSeq 2000 using a P3 kit to generate paired-end 150 bp reads (Illumina).

The quality of the raw sequence reads was reviewed using FastQC (V.0.11.3) (https://www.bioinformatics.babraham.ac.uk/projects/fastqc/). These were then aligned to an indexed human reference genome using BWA (V.0.7.12) (https://bio-bwa.sourceforge.net/).^28^ PCR duplicates were removed using Picard (V.2.5.0) (https://broadinstitute.github.io/picard/). Non-reference bases were identified and recorded in variant call format using the Genome Analysis Tool Kit (GATK) HaplotypeCaller (V.3.5) (https://gatk.broadinstitute.org).^29^ Prior to filtering, identified variants were annotated with functionally relevant biological information and observed population allele frequencies using the Variant Effect Predictor (VEP) (V.83).^30^

### Targeted sequencing of known AI genes (NHS)

Genes in the R340 (amelogenesis imperfecta) panel of the UK NHS National Genomic Test Directory were subject to hybridisation capture using a custom SureSelect reagent. Libraries were generated from 3 µg genomic DNA according to the manufacturer’s instructions (Agilent Technologies). Libraries were sequenced using a NovaSeq 6000 (Illumina). The resulting FASTQ files were processed and aligned as described above.

### Single molecule molecular inversion probes (smMIP) sequencing

smMIPs targeting the coding sequences of 19 genes (online supplemental table S4) implicated in non-syndromic AI were designed using MIPGEN (https://github.com/shendurelab/MIPGEN)^31^ and synthesised by Integrated DNA Technologies (IDT, Leuven, Belgium) at 100 nmol scale. Then, 100 ng genomic DNA was subjected to targeted capture and ligation using the smMIPs probe pool diluted to reach a ratio of 800 smMIPs copies for every one DNA molecule in the final capture reaction.^32^ Sequencing was carried out on a NextSeq 500 (Illumina) which generated paired-end 150 bp reads.

10.1136/jmg-2023-109510.supp1 Supplementary data

Data processing was performed using the MIPVAR pipeline (https://sourceforge.net/projects/mipvar/) which was modified for compatibility with local computing hardware. This enabled sample demultiplexing and the removal of unique molecular identifiers prior to ligation-arm and extension-arm processing using standard tools BWA (V.0.7.12), Picard (V.1.102.0) and the GATK HaplotypeCaller (V.3.2–2).

### Variant classification

The pathogenicity of the variants was assessed according to the American College of Medical Genetics and Genomics (ACMG) criteria using Franklin by Genoox (https://franklin.genoox.com/clinical-db/home).^33^ Allele frequencies were obtained from the Genome Aggregation Database V.2.1.1 (https://gnomad.broadinstitute.org/).^34^ Splicing predictions were generated using SpliceAI (https://spliceailookup.broadinstitute.org).^35^

### Sanger sequencing verification

Primers were designed using AutoPrimer3 (https://github.com/david-a-parry/autoprimer3) and synthesised by IDT. About 25 ng genomic DNA was amplified using Q5 High-Fidelity 2X Master Mix (NEB) according to manufacturer’s instructions. PCR products were purified using ExoSAP-IT (Applied Biosystems) then sequenced using BigDye Terminator V.3.1 chemistry on an ABI3130xl Genetic Analyser (Applied Biosystems). Electropherograms were analysed using SeqScape software V.2.5 (Applied Biosystems).

### Micro-computed tomography (µCT)

Intact teeth were analysed using a high resolution µ-CT SkyScan 1172 (Bruker, Belgium) scanner to quantify mineral density. Mineral density values were calculated relative to three hydroxyapatite standards of 0.25 and 0.75 g/cm^3^ (Bruker, Belgium) and 2.9 g/cm^3^ (Himed, USA). Fiji/ImageJ was used to analyse enamel density, with a pixel threshold above 2.0 g/cm^3^.

### Scanning electron microscopy (SEM)

Longitudinal mid buccal slices of teeth were obtained using an Accutom 10 cutting machine and diamond cutting wheel (Struers, Germany). After removing surface debris, slices were gold coated (Agar Scientific, Elektron Technology, UK). Imaging was performed by S-3400N (Hitachi, Japan) SEM.

## Results

### Cohort screening

**Figure 2:**
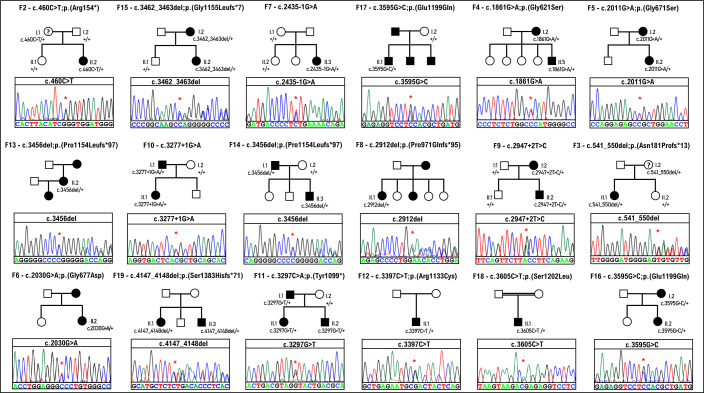
Pedigrees of 18 of the 19 families recruited for this study. The Sanger sequencing chromatogram from the proband from each family is displayed beneath each pedigree. Details of the family pedigree and variant identified in family F1 are displayed in online supplemental figure S2. A ‘?’ mark in the pedigree means ‘individuals with possible AI not clinically assessed’.

Genomic DNA from probands in a large cohort of apparently unrelated families with non-syndromic AI were investigated either by targeted smMIP screening, NHS diagnostic AI screening or WES. Variants identified were excluded if the CADD score was <15 or minor allele frequency was >0.001. The variant list for each case was then filtered further to include only candidate pathogenic variants in known or potential candidate AI genes. Where possible, additional filtering was performed based on family history. Probands from 19 families were identified as carrying potentially pathogenic variants in the *COL17A1* gene. In each case, this was the only variant in the known non-syndromic AI genes that met these criteria. Of these, inheritance in 12 families appeared dominant, while the mode of inheritance could not be determined in the remaining families due to incomplete clinical information about additional family members. Variant pathogenicity was assigned according to the ACMG criteria, with the outcome being pathogenic, likely pathogenic or a variant of unknown significance (VUS). All the suspected variants were re-sequenced by Sanger sequencing and their segregation with disease was checked in available family members (figure 2 and online supplemental figure S2).

### *COL17A1* variants

Probands in the 19 non-syndromic AI families displayed in figure 2 and online supplemental figure S2 were found to carry heterozygous, potentially pathogenic variants in *COL17A1*. Two variants were present in two families, with the remainder each occurring in only one family. None of the 17 variants described in this study were previously associated with AI. Only seven are present in the gnomAD database (table 1). Of the 17 variants, 6 are missense: c.1861G>A; p.(Gly621Ser), c.2011G>A; p.(Gly671Ser), c.2030G>A; p.(Gly677Asp), c.3397C>T; p.(Arg1133Cys), c.3595G>C; p.(Glu1199Gln), c.3605C>T; p.(Ser1202Leu); six result in frameshifts: c.340del; p.(Ser114Valfs*60), c.541_550del; p.(Asn181Profs*13), c.2912del; p.(Pro971Glnfs*95), c.3456del; p.(Pro1154Leufs*97), c.3462_3463del; p.(Gly1155Leufs*7) and c.4147_4148del; p.(Ser1383Hisfs*71); 3 are predicted to affect splice sites: c.2435–1G>A (acceptor loss score 0.99, acceptor gain score 0.92), c.2947+2T>C (donor loss score 0.98) and c.3277+1G>A (donor loss score 0.97); and 2 create premature termination codons (PTC): c.460C>T; p.(Arg154*) and c.3297C>A; p.(Tyr1099*).

**Table 1:** Details of *COL17A1* variants reported in this study

Family ID	ACMG criteria	Variants			CADD score	gnomAD frequency
		Genomic nomenclature	Transcript nomenclature	Predicted protein nomenclature		
F1	P (PVS1, PM2, PP5)	g.105833981del	c.340del	p.(Ser114Valfs*60)	32.0	0.00001591
F2	P (PP1, PP4, PVS1, PM2, PP5)	g.105831793G>A	c.460C>T	p.(Arg154*)	36.0	0.000003977
F3	P (PP1, PP4, PVS1, PM2)	g.105830245_105830254del	c.541_550del	p.(Asn181Profs*13)	32.0	Absent
F4	LP (PP1, PP3, PP4, PM2)	g.105812867C>T	c.1861G>A	p.(Gly621Ser)	23.9	Absent
F5	LP (PP1, PP3, PP4, PM2)	g.105811266C>T	c.2011G>A	p.(Gly671Ser)	26.3	0.0001135
F6	LP (PP3, PP4, PM2)	g.105811247C>T	c.2030G>A	p.(Gly677Asp)	26.1	Absent
F7	LP (PP1, PP4, PVS1, PM2)	g.105803340C>T	c.2435–1G>A	p.?	34.0	Absent
F8	P (PP4, PVS1, PM2)	g.105798865del	c.2912del	p.(Pro971Glnfs*95)	33.0	Absent
F9	LP (PP1, PP4, PVS1, PM2)	g.105798827A>G	c.2947+2T>C	p.?	30.0	Absent
F10	P (PP1, PP4, PVS1, PM2, PP5)	g.105796802C>T	c.3277+1G>A	p.?	27.7	0.00006312
F11	P (PP1, PP4, PVS1, PM2)	g.105796371G>T	c.3297C>A	p.(Tyr1099*)	36.0	Absent
F12	VUS (PP4, PM2)	g.105796271G>A	c.3397C>T	p.(Arg1133Cys)	33.0	Absent
F13	P (PP4, PVS1, PM2)	g.105795287del	c.3456del	p.(Pro1154Leufs*97)	20.6	0.000008021
F14	P (PP1, PP4, PVS1, PM2)	g.105795287del	c.3456del	p.(Pro1154Leufs*97)	20.6	0.000008021
F15	P (PP1, PP4, PVS1, PM2)	g.105795277_105795278del	c.3462_3463del	p.(Gly1155Leufs*7)	33.0	Absent
F16	LP (PP1, PP4, PS4, PM2)	g.105795045C>G	c.3595G>C	p.(Glu1199Gln)	25.1	Absent
F17	LP (PP1, PP4, PS4, PM2)	g.105795045C>G	c.3595G>C	p.(Glu1199Gln)	25.1	Absent
F18	VUS (PP4, PM2)	g.105795035G>A	c.3605C>T	p.(Ser1202Leu)	27.0	0.00002800
F19	P (PP1, PP4, PVS1, PM2)	g.105793715_105793716del	c.4147_4148del	p.(Ser1383Hisfs*71)	34.0	Absent

ACMG scoring criteria: PP1: segregation data pathogenic supporting; PP4: phenotype pathogenic supporting; PS4: case control studies pathogenic strong; PVS1: effect on protein pathogenic very strong; PM2: population data pathogenic moderate; PP5: reputable source data pathogenic supporting.

Nomenclature is reported according to *COL17A1* transcript NM_000494.4 and chromosome 10 of human reference genome build hg19.

CADD V.1.3, combined annotation dependent depletion; gnomAD V.2.1.1, genome aggregation database; LP, likely pathogenic; P, pathogenic; VUS, variant of unknown significance.

All the stop and frameshift variants identified are classified as pathogenic, while the three splice site variants are classified as pathogenic or likely pathogenic. Among the six missense variants three are glycine substitutions, and these are classified as likely pathogenic. Of the three remaining missense variants, one, p.(Glu1199Gln), was initially classed as a VUS, but is absent from gnomAD and was observed to co-segregate in two families reported here, leading to reclassification as likely pathogenic. The remaining two non-glycine missense variants are currently classified as variants of unknown significance (VUS). All missense variants identified are in the extracellular domain of the protein (figure 1).

### Oral clinical phenotype

**Figure 3:**
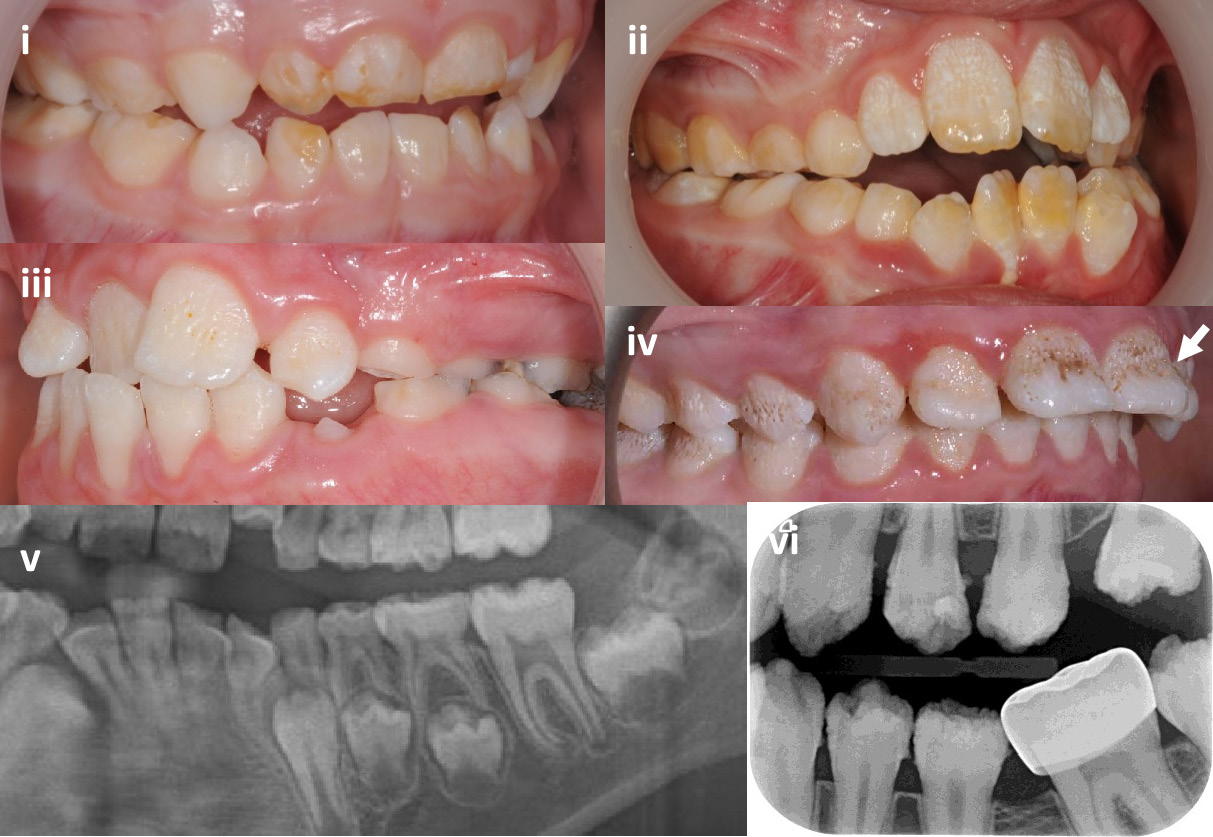
Intraoral images and dental radiographs illustrating the variation in enamel phenotypes associated with heterozygous *COL17A1* variants in primary and secondary teeth. (i) Primary tooth enamel changes can be minimal and easily missed and are primarily characterised by hypomaturation changes with subtle surface focal pitting (F10). (ii) A predominantly hypomaturation AI phenotype with some surface irregularities (F9). (iii) Surface pits and other irregularities are the clinically dominant feature, on a background of hypomaturation (F4). (iv) Hypomaturation enamel is combined with more exaggerated surface pits merging into grooves with mid-third crown regional hypoplasia (arrow) (F3). (v) Section of an orthopantomogram of a mixed dentition illustrating near normal enamel thickness with a normal difference in radiodensity between the enamel and the supporting dentine (F15). (vi) Intraoral radiograph illustrating near normal enamel thickness, but with enamel irregularities and a lesser difference in radiodensity between enamel and dentine than would be expected (F5). Further clinical images and dental radiographs are included in online supplemental figure S4.

All families presented as isolated AI with no history of co-segregating health issues. Variability in the clinical AI phenotype was evident, with features that reflected enamel qualitative and quantitative changes (figure 3 and online supplemental figure S4).

Clinical enamel changes in the primary dentition were minimal and could be easily overlooked. Hypomaturation changes were more evident where there had been some post-eruptive enamel loss. Focal surface pitting was subtle.

In the secondary dentition, there was generalised, but clinically variable enamel hypomaturation characterised by white to yellow/brown colouration and greater enamel opacity than expected. Surface irregularities were also variable, with distinct, deep pits that in some instances were obvious due to extrinsic staining or formed linear, vertical defects in the most pronounced cases. Shallow surface irregularities were also present in some teeth. Regional enamel hypoplasia involving the middle third of the labial aspect of anterior teeth was observed in some cases. Dental radiographs confirmed that enamel thickness was for the most part within expected normal limits. A clear distinction between the radiodensity of enamel compared with the supporting dentine confirmed that any reduction in enamel mineralisation was at the mild end of the spectrum, consistent with the clinical hypomaturation phenotype. Post-eruption enamel loss was not obviously exaggerated.

Tooth root morphology including pulp spaces was within expected normal limits with no taurodontism. No oral mucosal or other oral cavity changes were evident.

### Laboratory analysis of teeth

**Figure 4:**
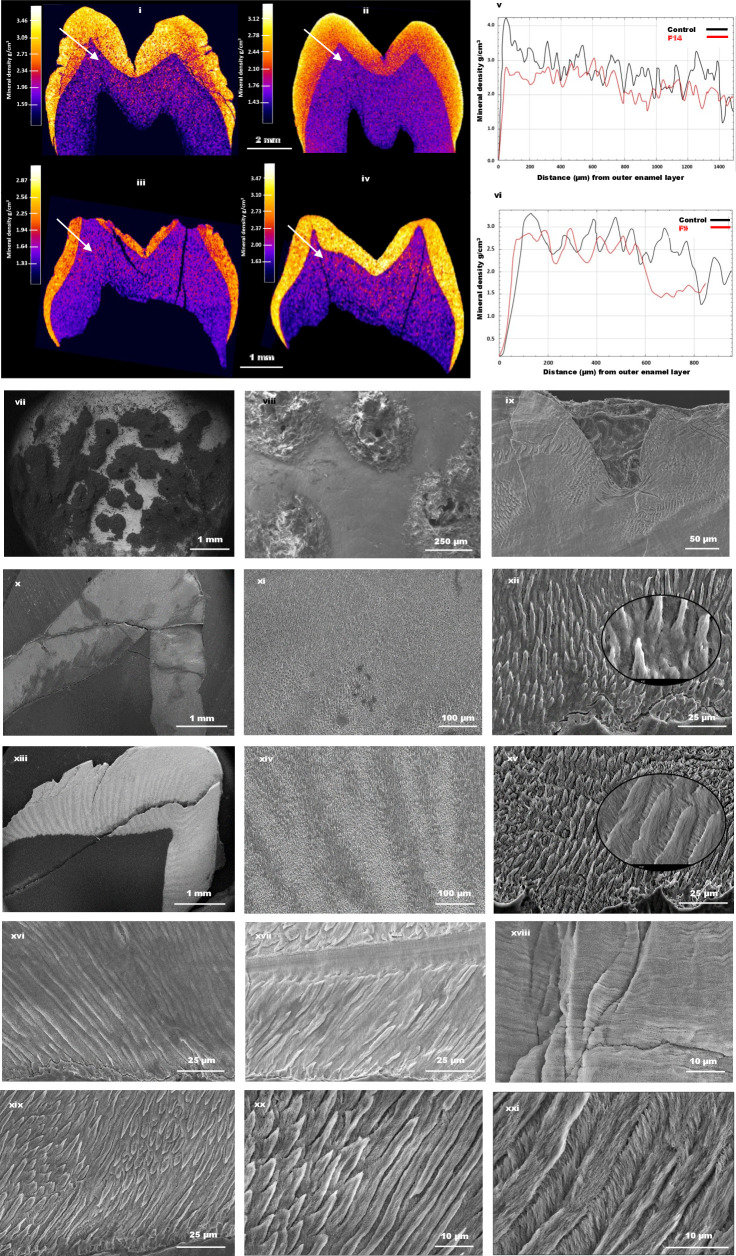
Laboratory analysis of teeth. (i) Micro-computed tomography (µ-CT) imaging of a permanent upper first premolar tooth from the proband of F14 and (iii) a primary upper molar tooth from the affected individual from F9. Panels (ii) and (iv) have images from corresponding control teeth. No significant differences were observed in average enamel mineral density (EMD) between affected and control samples. F14 and its corresponding control had EMD values of 2.58 and 2.60 g/cm^3^, respectively, while F9 and its corresponding control were 2.40 and 2.52 g/cm^3^, respectively. (v–vi) Line graphs showing the distribution of mineral density from the enamel surface to the dental enamel junction (DEJ), as shown by the arrows in (i–iv). Affected samples (red) lack high mineral density at the surface, as opposed to the control teeth (black). Scanning electron microscopy (SEM) images of the enamel in F14 (vii–viii) and F9 show clear pitting extending towards DEJ.^51^ SEM of enamel from F14 (x–xii) shows generally disrupted and poorly formed prismatic microstructure compared with the corresponding control teeth in the images (xiii–xv), respectively. SEM images of the enamel prism in F9 (xvi–xviii) appears as a stack of lamellae, with patches of fused rod-interrod regions hard to distinguish between them. However, enamel from corresponding controls show distinguishable rod interrod regions (xix–xxi).

Upper primary molar teeth from affected members of families F9 and F14 were analysed by three-dimensional µCT and SEM and compared with the relevant control teeth (figure 4, online supplemental figure S3). µCT revealed near normal enamel volume in the affected teeth, but they lacked a hard outer enamel layer and mineral density gradation from higher to lower moving from the outer enamel towards the dental enamel junction (DEJ), by comparison with the control teeth. µCT also revealed a pitted and uneven enamel surface in the probands’ teeth, in both primary and permanent dentitions, confirmed by SEM analysis, which showed the presence of pitting, and disruption of the enamel layers appearing as a stack of lamellae, with patches of fused rod-interrod regions hard to distinguish between them (figure 4 and online supplemental figure S3).

### Wider clinical phenotype

None of the affected individuals described here were noted to have skin or mucosal abnormalities, corneal problems or any other associated conditions. However, all were recruited in dental clinics as cases of non-syndromic AI and have not been examined by other clinical specialists for subtle skin or corneal presentations.

## Discussion

Here, we report 15 pathogenic/likely pathogenic heterozygous *COL17A1* variants as the likely cause of non-syndromic AI in 17 probands, as well as 2 further cases with VUSs that may also be causative. This greatly increases the previous tally of six, within an increasingly clear context that *COL17A1* variants are a frequent and under recognised cause of dominantly inherited AI. The AI phenotype observed is consistent with the limited clinical images, radiographs and other data in the peer-reviewed literature from carriers in JEB families who are heterozygous for *COL17A1* variants.

AI due to heterozygous *COL17A1* variants has been linked to the Witkop classification type 1a pitted hypoplastic AI.^26 36^ Witkop described hypoplastic, pitted autosomal dominant type enamel with pits from pinpoint to pinhead size primarily on labial or buccal surfaces of permanent teeth, often arranged in rows and columns, but with comment that some teeth may appear normal in both dentitions. The Witkop classification evolved over time but remained primarily clinically descriptive, with patterns of inheritance included in some instances. Data presented here highlight the advantages of switching to classification where genetic diagnosis has primacy and is correlated to the clinical enamel phenotype that can vary within certain parameters. This is also with recognition that enamel does not have cellular capacity for repair and that the enamel phenotype is altered by post-eruption changes. The enamel present is generally well mineralised but shows disrupted enamel rod morphology, which can be expected to adversely impact enamel functional longevity. Teeth from individuals with JEB due to biallelic *COL17A1* variants were not available for comparative analysis. In summary, in this series affected enamel has hypomaturation characteristics with variable focal hypoplasia (pits and indentations) and in some instances, partial regional hypoplasia of the middle third of the tooth enamel.

The profound adverse impact of JEB on the affected individuals and their families has driven our understanding of how the condition is caused by pathological biallelic variants in *COL17A1*, *LAMA3*, *LAMB3*, *LAMC2*, *ITGA6*, *ITGB4* and *ITGA3*. According to the England and Wales EB database, JEB prevalence is around 1 per million, with most pathogenic variants detected in *LAMB3* (40%–50%), followed by *LAMC2* (15%–20%) and *LAMA3* (10%–15%), with only a small proportion (5%–10%) in *COL17A1* (John McGrath, personal communication). By contrast, the association of AI with heterozygous variants in these genes in families with dominant inheritance and no history of JEB, are less obviously presented in the published literature, which also fails to clarify whether affected individuals are carriers for JEB. While all individuals with JEB have AI (or enamel hypoplasia), there are very few reports of AI in carriers of JEB due to *COL17A1* variants, and it remains unclear what proportion of carriers will manifest enamel or corneal abnormalities.^21 37^ Assuming 1 in 10 million people have JEB due to *COL17A1* variants, Hardy-Weinberg equilibrium would predict a carrier frequency of approximately 1 in 1600, not inconsistent with published estimates of the frequency of AI,^38 39^ especially given that many such individuals may have been considered to have enamel hypoplasia or enamel opacities rather than inherited AI. This highlights two important related points where a molecular diagnosis can inform clinical decision-making. First, distinguishing between more subtle forms of AI and other enamel development defects. Second, that dental changes offer an opportunity to identify carrier status for JEB in families with no history of this condition.

If it is assumed that all JEB carriers have AI, then one might expect cases of AI due to variants in *LAMB3*, *LAMC2* and *LAMA3* to be more common than those with *COL17A1* variants, given the frequency of the different forms of JEB. Variants in all three genes have been reported in patients with AI in the literature but only in a handful of cases for each,^40 41^ while our findings show that variants in *COL17A1* are a relatively common cause of AI. It is therefore evident that further research is needed into the link between dominant AI and recessive JEB due to *COL17A1* variants.

We identified missense, PTC, frameshift and splice site variants in both the endo-domains and the ecto-domains of the protein. Fifteen of the variants described are novel, while two have been previously reported as pathogenic in JEB but not in isolated AI. Patients in this study were not reported to have any associated skin or corneal problems but have not been examined by dermatologists or cornea specialists, meaning that subtle versions of either condition could potentially have been overlooked. We wanted to understand whether the *COL17A1* variants associated with AI differ from those that cause JEB. By combining a literature search on the NCBI database (https://pubmed.ncbi.nlm.nih.gov/) with data from the HGMD professional database (accessed 30 March 2023),^42^ we identified 232 *COL17A1* variants reported to cause JEB (online supplemental table S1). The distribution of mutations and mutation types in JEB and AI are similar (online supplemental figure S1). Variants c.460C>T; p.(Arg154*) and c.1861G>A; p.(Gly621Ser), reported here as causing AI, and variant c.1745–2A>C, c.2407G>T; p.(Gly803*) and c.3327del; p.(Pro1110Argfs*21) reported to cause AI by Prasad and colleagues,^25^ have also been identified as pathogenic in JEB,^5 43 44^ showing there is overlap in the underlying genetic basis of these conditions.

Only three *COL17A1* variants have been reported in the literature as causing the corneal disease ERED (online supplemental table S3). None of these have been implicated in AI or JEB. The nonsense variant, p.(Arg154*), detected in an AI proband in this study and in JEB, was reported in ClinVar (VCV000931124.3) as causing autosomal dominant ERED. However, no further evidence was provided, and this result remains unpublished at the time of writing, meaning this should be considered unconfirmed at this stage. It is unknown if individuals with ERED due to dominant *COL17A1* variants have AI, but the expectation until demonstrated otherwise is that they will, although many of these patients will not have been thoroughly examined by dentists.

Most variants associated with JEB, AI and ERED are frameshift, splice or PTC. The consequences of these variants have not been determined experimentally, but it seems likely that they will be subject to nonsense mediated decay,^45^ meaning no functional protein is produced from those alleles. Many of the individuals with JEB due to *COL17A1* variants are homozygous for such variants, meaning their phenotype is in effect the result of complete knockout of *COL17A1*. It therefore seems likely that many with JEB suffer from near-complete loss of Collagen XVII protein. Since two of the PTCs implicated in JEB were also found in AI, and given the unconfirmed report of one of the same variants in an ERED case, it therefore seems likely that AI and ERED due to heterozygous *COL17A1* pathogenic variants is caused, at least in some cases, by haploinsufficiency. Further evidence for this disease mechanism comes from the work of Yuen and colleagues,^37^ who used immunofluorescence staining with antibodies targeted to mouse Col17 to show reduced basement membrane zone and apical–lateral staining in skin from both JEB patients and carriers compared with control skin.

A proportion of the *COL17A1* variants observed in individuals with JEB, AI and ERED are amino acid substitutions. These may also be functional knockouts, but an alternative disease mechanism has been proposed in some of these cases. Missense variants, and most commonly glycine substitutions, have been reported to be associated with milder JEB phenotypes.^17^ Substitution of glycine residues within the ectodomain, and particularly within the COL15 collagenous sequence (figure 1), is thought to destabilise the collagenous triple helix, making the protein unstable, with the mutated protein predicted to exert a dominant negative effect on the wild-type protein.^46 47^ Interestingly, of the six missense variants reported here in patients with AI, three were glycine substitutions in the COL15 region.

An interesting case describes a patient with JEB who is a compound heterozygote for glycine substitution p.Gly627Val within the COL15 domain and frameshift insertion c.3514ins25 within the COL6 domain.^20^ The proband had an abnormal dentition, with complete loss of all teeth by age 14. The proband’s daughter, who is a heterozygous carrier of the p.Gly627Val variant, showed no skin abnormalities but had extensive enamel hypoplasia and pitting. The proband’s granddaughter, who was also a carrier of the p.Gly627Val variant, manifested dental abnormalities and trauma-induced skin blistering, especially around the knees. The authors concluded that p.Gly627Val has a dominant negative effect on the collagen XVII protein, causing autosomal dominant JEB in the granddaughter.^20 48^ As well as providing further evidence of a dominant negative disease mechanism and of overlap between the *COL17A1* variants causing JEB and AI, this case illustrates the importance of a multidisciplinary approach to the clinical care of such patients.

These findings have significant implications for future care of individuals and their families with diagnoses of JEB, AI or ERED due to pathogenic variants in *COL17A1*. Further studies are needed to better understand links between these conditions, but it seems likely that there is overlap between carrier status for JEB and a diagnosis of AI or ERED when they result from heterozygous *COL17A1* pathogenic variants. This may not have been fully appreciated by disparate groups of clinicians treating each condition in isolation. The mucocutaneous lesions of JEB are generally so severe that corneal or dental problems may not have been prioritised in patients and could have been overlooked in their carrier parents or siblings. ERED manifests at around 5 years, but may resolve by the early 20s, meaning many adults with the condition are without symptoms. AI may be dismissed by non-experts as resulting from poor dental hygiene, especially in children with EB, who have considerable difficulty in maintaining oral hygiene for multiple reasons.^49^

To summarise, we identified 17 families with AI due to pathogenic/likely pathogenic heterozygous variants in the *COL17A1* gene, and a further 2 families with variants of unknown significance in *COL17A1* that may also be pathogenic. These findings suggest that the significance of *COL17A1* variants as a cause of AI has not been fully appreciated and this may in fact be a relatively common form of dominantly inherited AI. We detail the spectrum of the enamel phenotype observed and review all the pathogenic *COL17A1* variants known to cause AI. A comparison with those causing the recessive skin disorder JEB suggests they are similar in mutation type and distribution, and there is also direct overlap between the variants implicated in both conditions, and possibly in a third, the dominantly inherited corneal disorder ERED. People with AI or ERED due to heterozygous *COL17A1* variants should be considered carriers for JEB. Furthermore, these results highlight the need for a multidisciplinary approach to the care of families and individuals with JEB, including carriers, and those with dominant AI or ERED due to *COL17A1* variants.

## Data Availability

All data relevant to the study are included in the article or uploaded as supplementary information.
