# Supplementary material for: Heterozygous COL17A1 variants are a frequent cause of amelogenesis imperfecta
Source: J Med Genet. 2023 Nov 18;61(4):347–55. doi: 10.1136/jmg-2023-109510 (PMC10982616; doi:10.1136/jmg-2023-109510)

Supplementary Figure S4: Representative clinical images and dental radiographs for individuals with heterozygous *COL17A1* variants. Some teeth have been restored.

F2: c.460C>T:p.(Arg154*). Mixed primary and secondary dentitions. The enamel is characterised by hypomaturation with patchy variations in colour (white to brown) accompanied by greater opacity than normally expected. The enamel surface is irregular without obvious pitting. The underlying *COL17A1* variant is a known cause of JEB when homozygous.

F3: c.541_550del:p.(Asn181Profs*13). Secondary dentition. The enamel is characterised by hypomaturation with multiple surface pits that in places have contributed to linear defects, particularly in the middle third of the upper incisor teeth where there is regional hypoplasia. By comparison to the middle third, the incisal third of the labial crown surface has minimal, if any hypoplasia.

F4: c.1861G>A:p.(Gly621Ser). Mixed dentition. The enamel is characterised by mild hypomaturation with widespread surface irregularities including pits evident on the surface of the secondary teeth. There is mild hypoplasia of the mid-third of the labial surface of the upper central incisor teeth and minor morphological changes to the crown shapes of the secondary premolar teeth. The underlying *COL17A1* variant is a known cause of JEB when homozygous.

F5: c.2011G>A:p.(Gly671Ser). Secondary dentition. The enamel is characterised by hypomaturation and multiple surface irregularities including focal pits. There are minor morphological changes to the crown shapes of the secondary premolar teeth. Radiographs identify a clear distinction in radiodensity between enamel and dentine, consistent with a hypomaturation phenotype. There are also irregularities in the enamel morphology consistent with the clinical images.

F6: c.2030G>A:p.(Gly677Asp). Mixed dentition. The enamel is characterised by hypomaturation with variable colour changes and multiple surface irregularities. The secondary dentition first molar teeth appear more obviously affected than other teeth. There is mild hypoplasia of the mid-third of the labial surface of the upper central incisor teeth and minor morphological changes to the crown shapes of the secondary premolar teeth.

F7: c.2435-1G>A:p.? Mixed dentition. The enamel is characterised by hypomaturation with multiple surface pits and regional hypoplasia in the middle third of the upper incisor teeth. By comparison to the middle third, the incisal third of the labial crown surface has minimal, if any hypoplasia. There are minor morphological changes to the crown shapes of the secondary dentition premolar teeth that have a consistent pattern with those observed in the upper central incisor teeth.

F9: c.2947+2T>C:p.? Mixed dentition. The enamel is characterised by hypomaturation with patchy variations in colour (white to brown) accompanied by greater opacity than normally expected. widespread mild surface irregularities evident on the surface of the secondary teeth.

F10: c.3277+1G>A:p.? Primary dentition. The enamel is predominantly characterised by hypomaturation with only minimal surface irregularities.

F11: c.3297C>A:p.(Tyr1099*). Secondary dentition. The enamel is characterised by hypomaturation with widespread mild surface irregularities and focal pits that have a variable distribution over the crown surfaces.

F12: c.3397C>T:p.(Arg1133Cys). Secondary dentition. Limited information is available. The attrition to many of the permanent teeth illustrates an enamel thickness within expected limits.

F14: c.3456del:p.(Pro1154Leufs*97). Secondary dentition. The enamel is characterised by hypomaturation with patchy variations in colour (white to yellow/brown) and multiple surface irregularities.

Supplementary Figure S4 *cont*: Representative clinical images and dental radiographs for individuals with heterozygous *COL17A1* variants. Some teeth have been restored.

F15: c.3462_3463del:p.(Gly1155Leufs*7). Secondary dentition. The radiograph illustrates enamel thickness being within expected limits and a clear distinction in radiodensity between enamel and dentine, consistent with a hypomaturation phenotype.

F18: c.3605C>T:p.(Ser1202Leu). Secondary dentition. The enamel is characterised by hypomaturation and multiple surface irregularities without obvious focal pits. There are minor morphological changes to the crown shapes of the secondary premolar teeth. A panoramic radiograph of the mixed dentition identifies a clear distinction in radiodensity between enamel and dentine, consistent with a hypomaturation phenotype.

No clinical images or dental radiographs were available for the following families. The clinical phenotype in all families was described as pitted hypoplastic AI.

F8: c.2912del:p.(Pro971Glnfs*95) - Pathogenic
F13: c.3456del:p.(Pro1154Leufs*97) - Pathogenic
F16: c.3595G>C:p.(Glu1199Gln) - Likely Pathogenic
F17: c.3595G>C:p.(Glu1199Gln) - Likely Pathogenic
F19: c.4147_4148del:p.(Ser1383Hisfs*71) - Pathogenic

F2 - c.460C>T:p.(Arg154*) - Pathogenic (Condrat et al 2018)

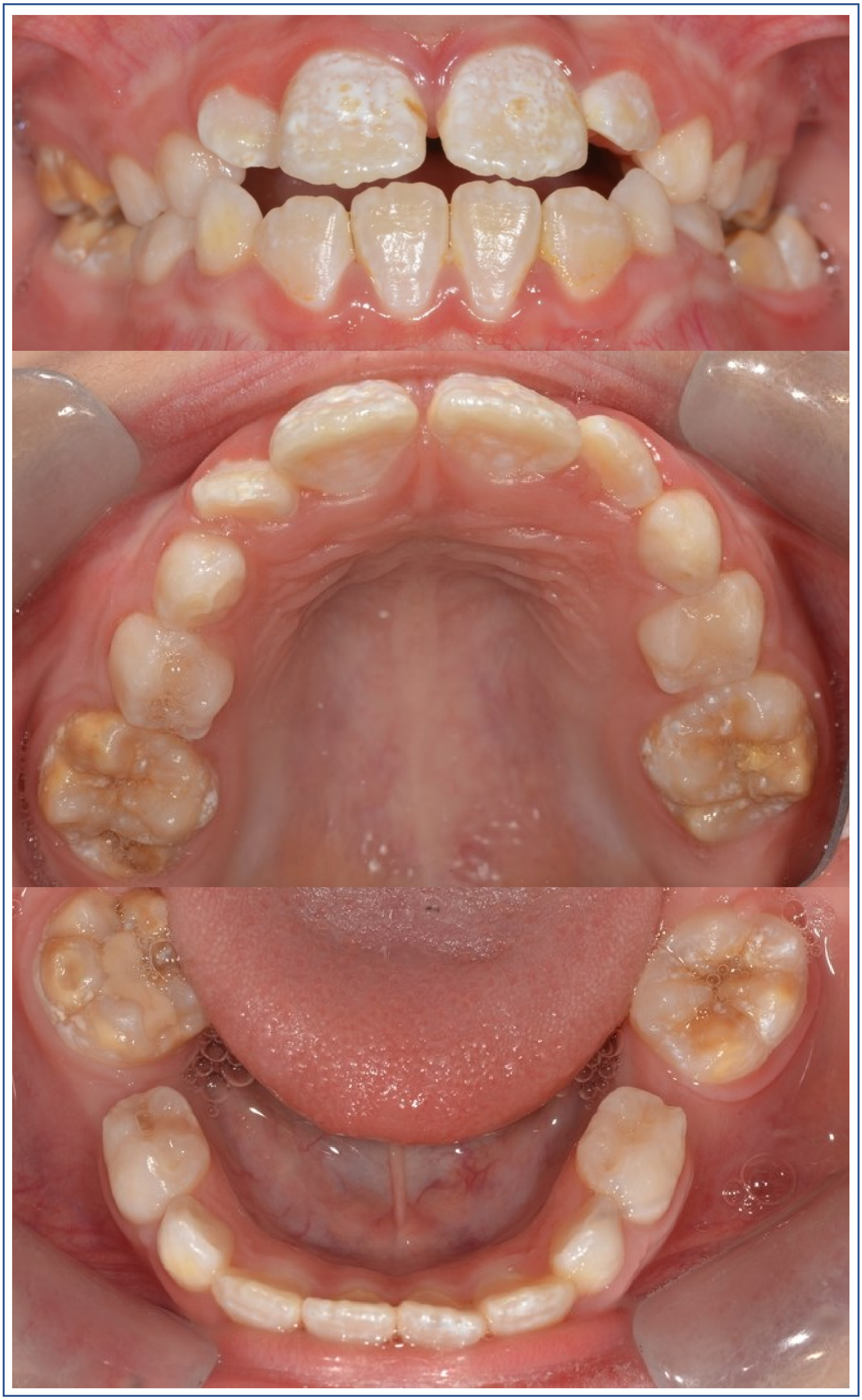

F2 continued - c.460C>T:p.(Arg154*) - Pathogenic (Condrat et al 2018)

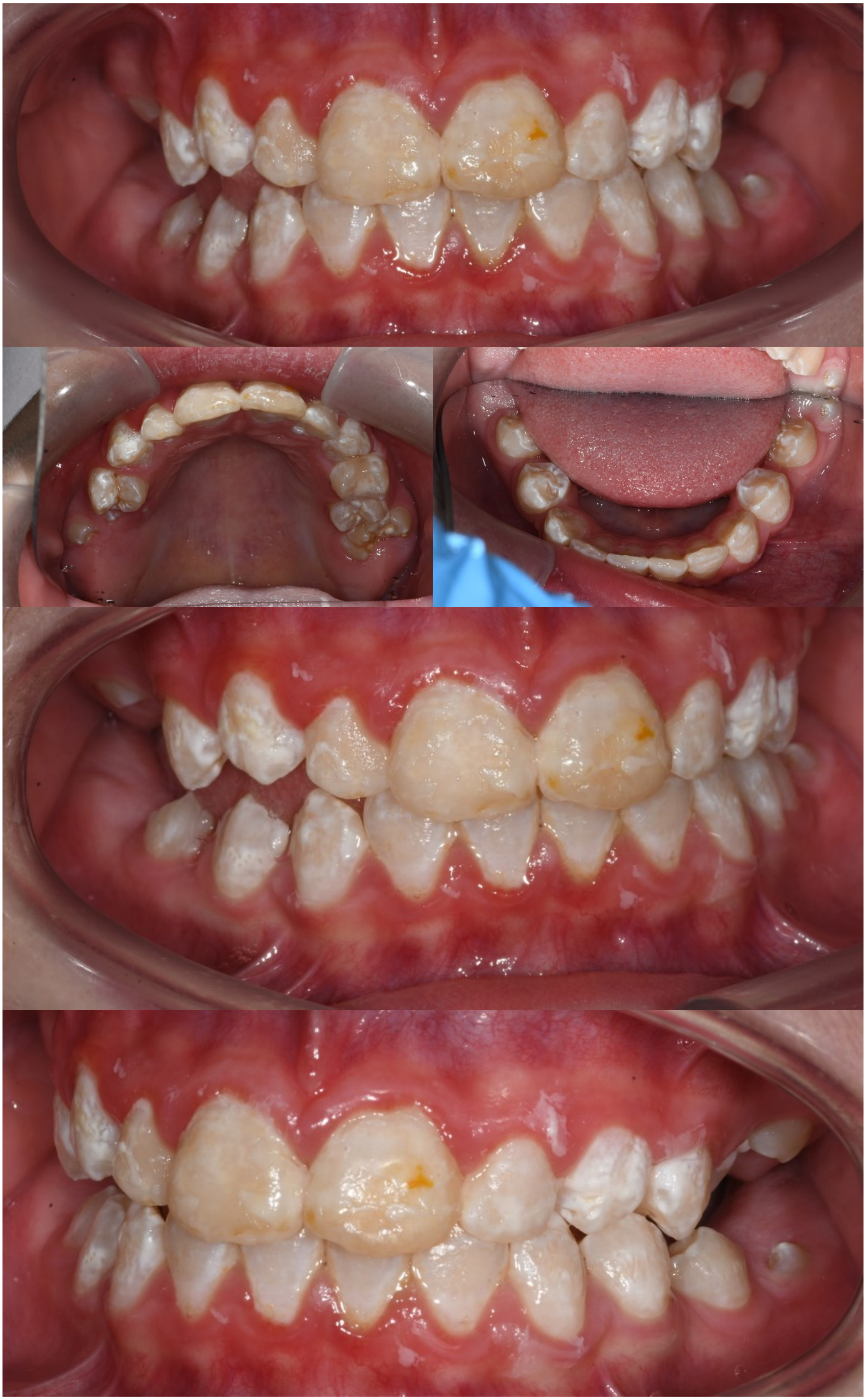

F3 - c.541_550del:p.(Asn181Profs*13) - Pathogenic

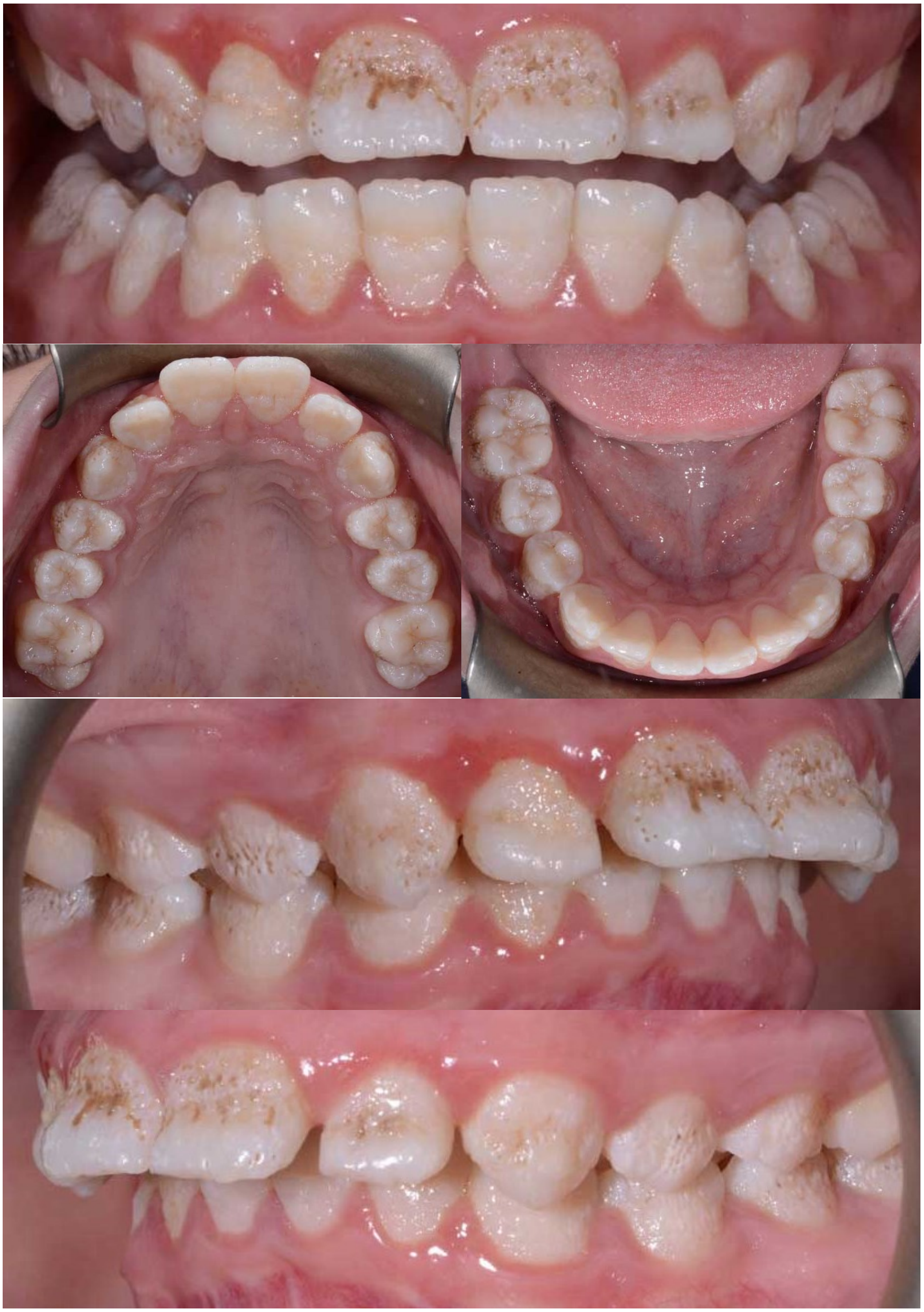

F4 – c.1861G>A:p.(Gly621Ser) - Likely Pathogenic

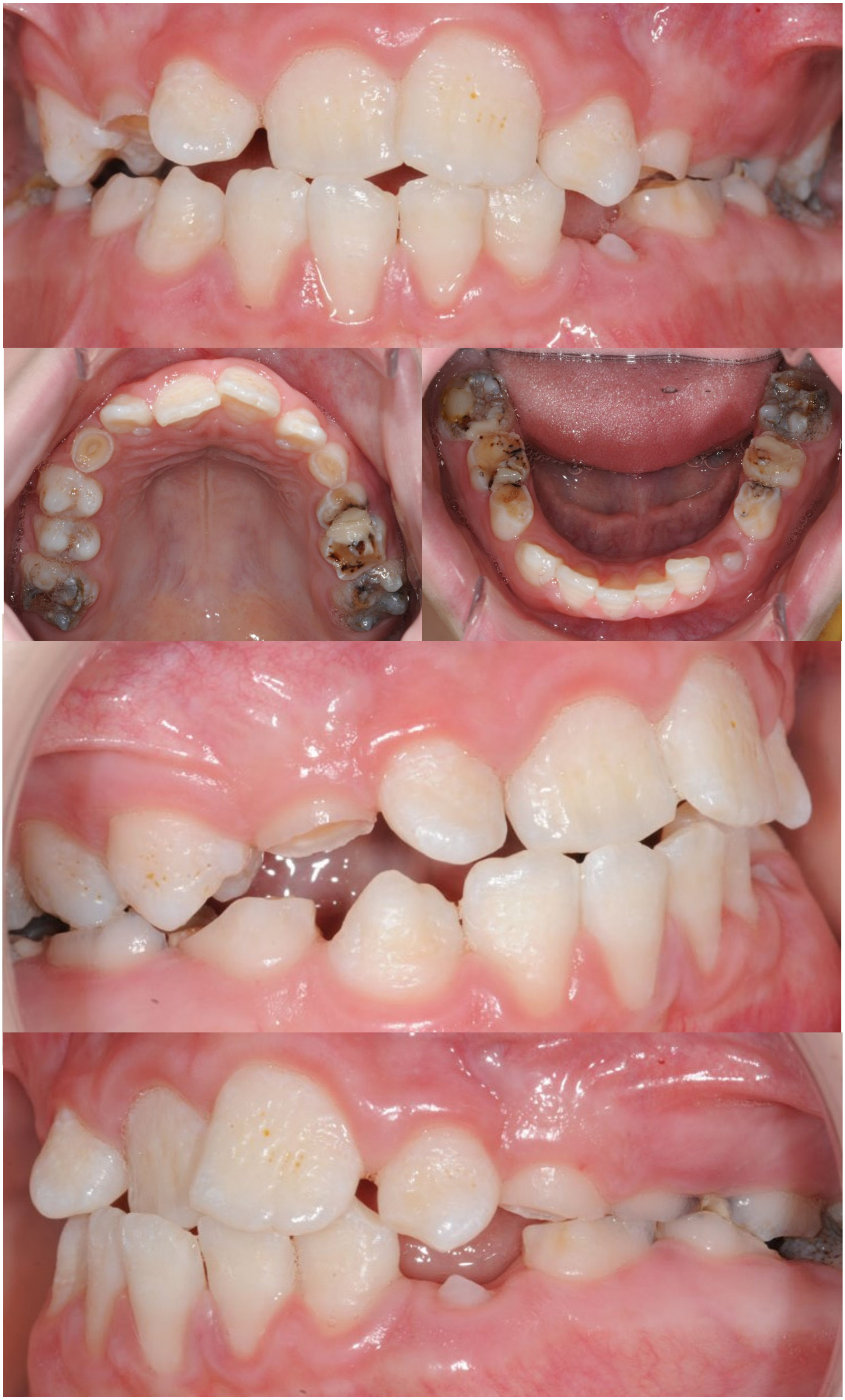

F5 - c.2011G>A:p.(Gly671Ser) - Likely Pathogenic

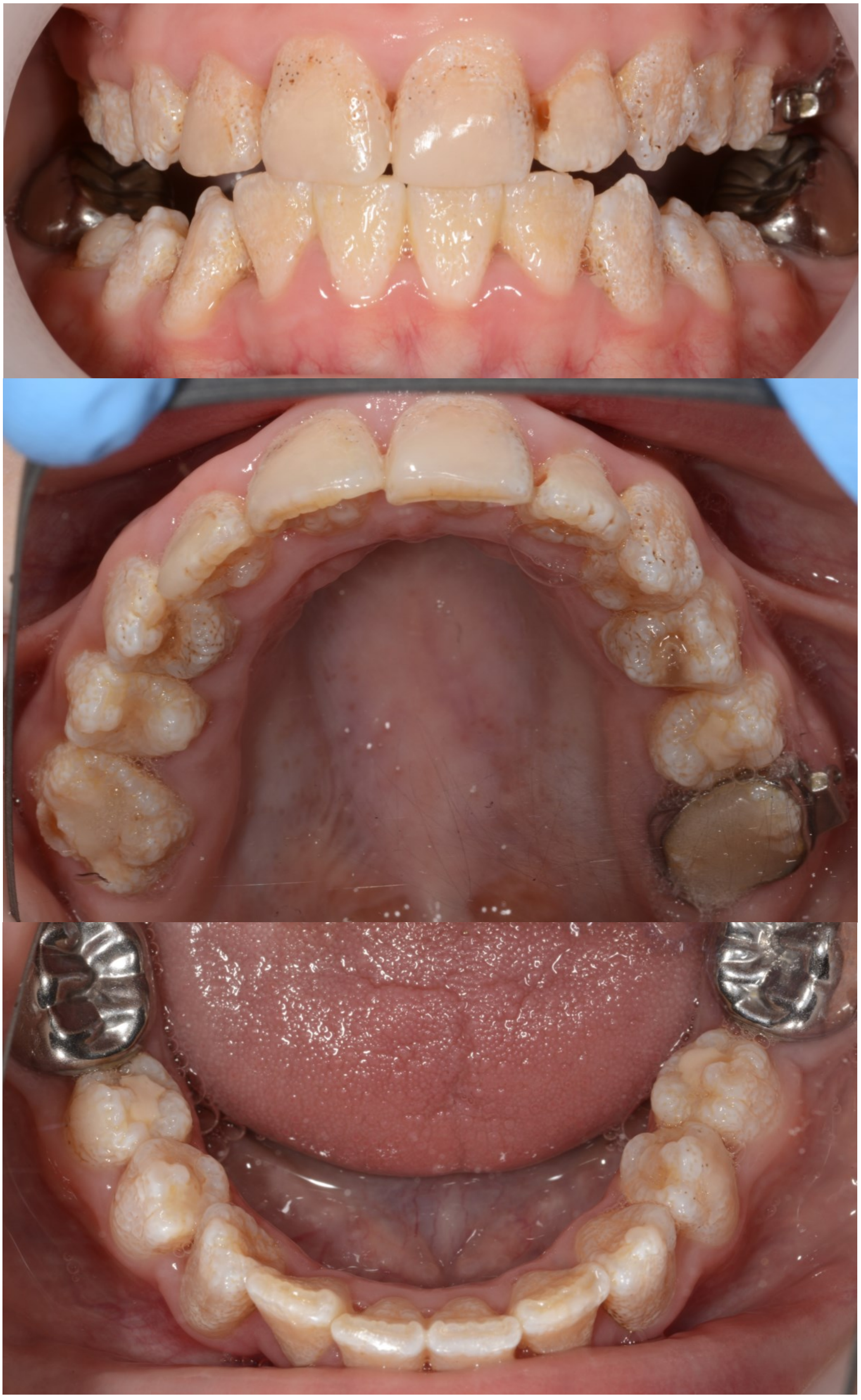

F5 - c.2011G>A:p.(Gly671Ser) - Likely Pathogenic

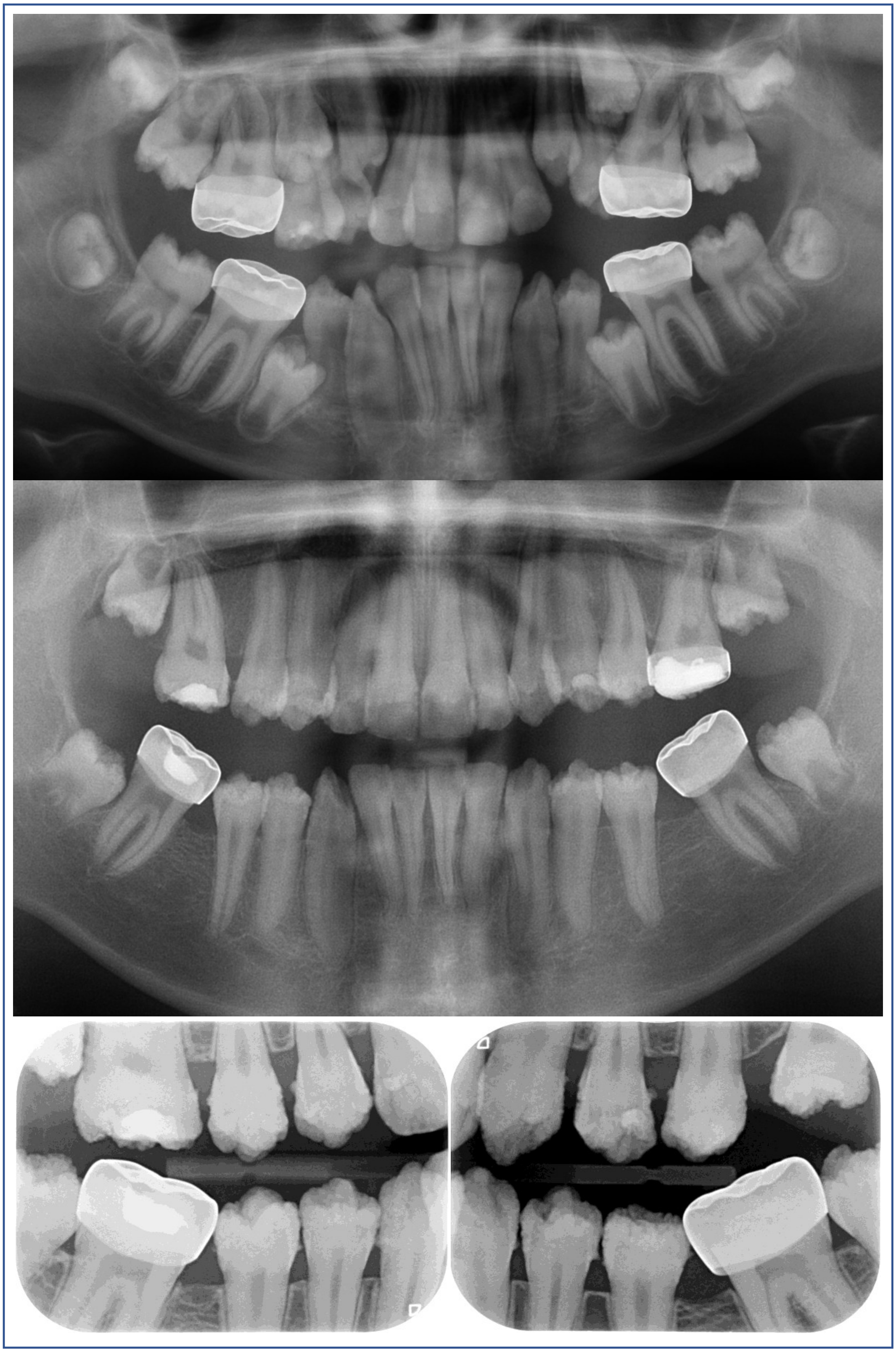

F6 - c.2030G>A:p.(Gly677Asp) - Likely Pathogenic

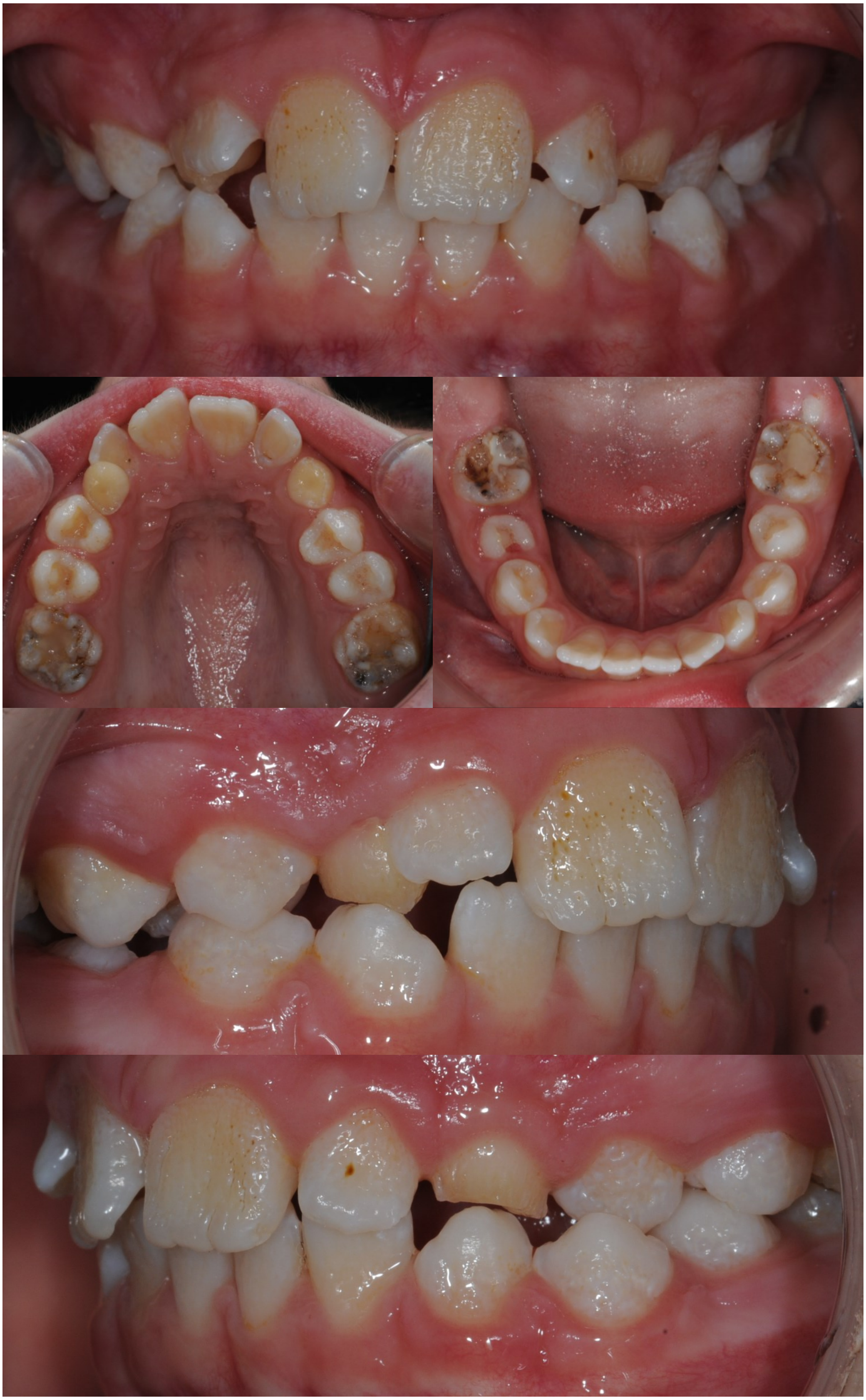

F7 - c.2435-1G>A:p.? - Likely pathogenic

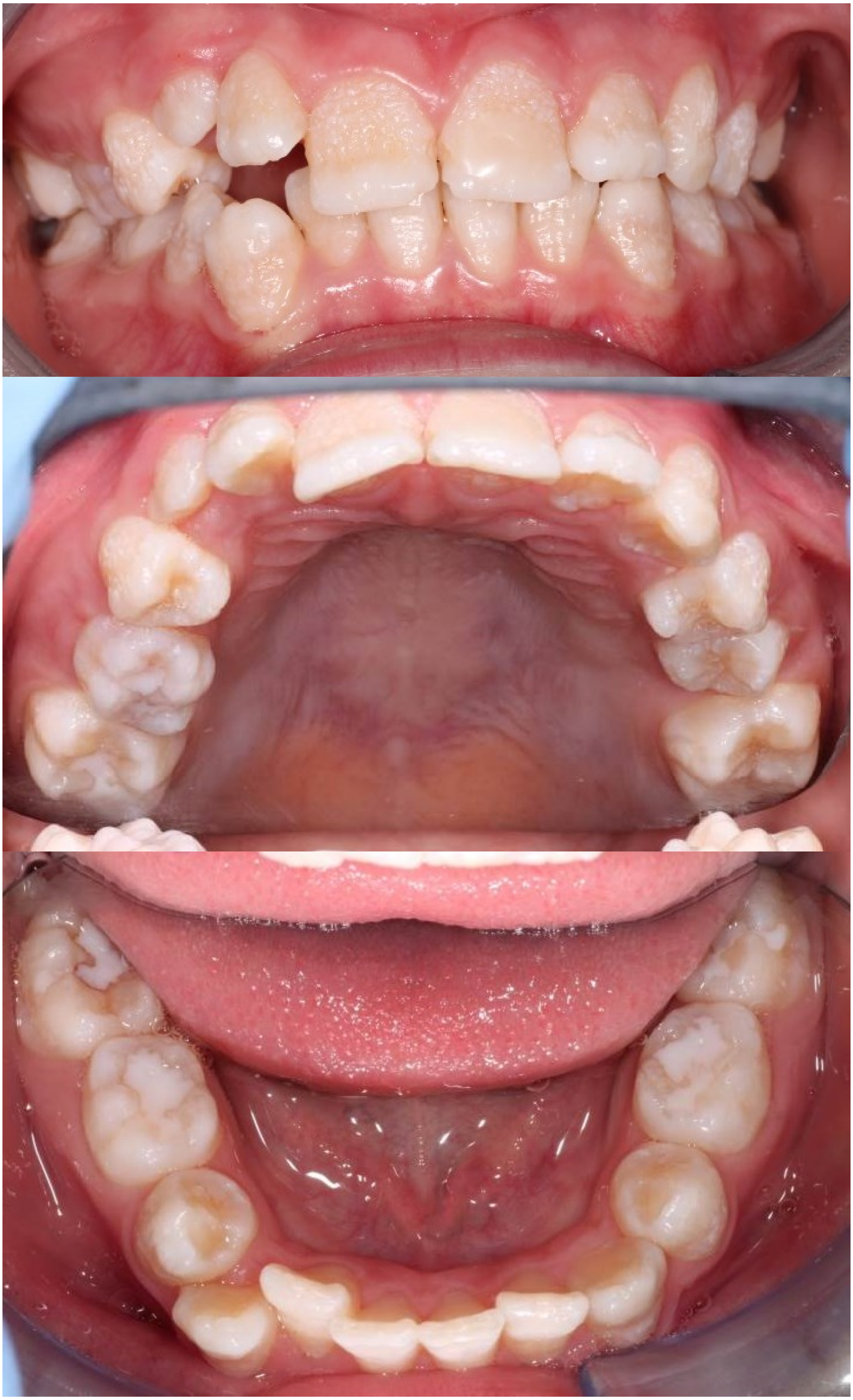

F9 - c.2947+2T>C:p.? - Likely Pathogenic

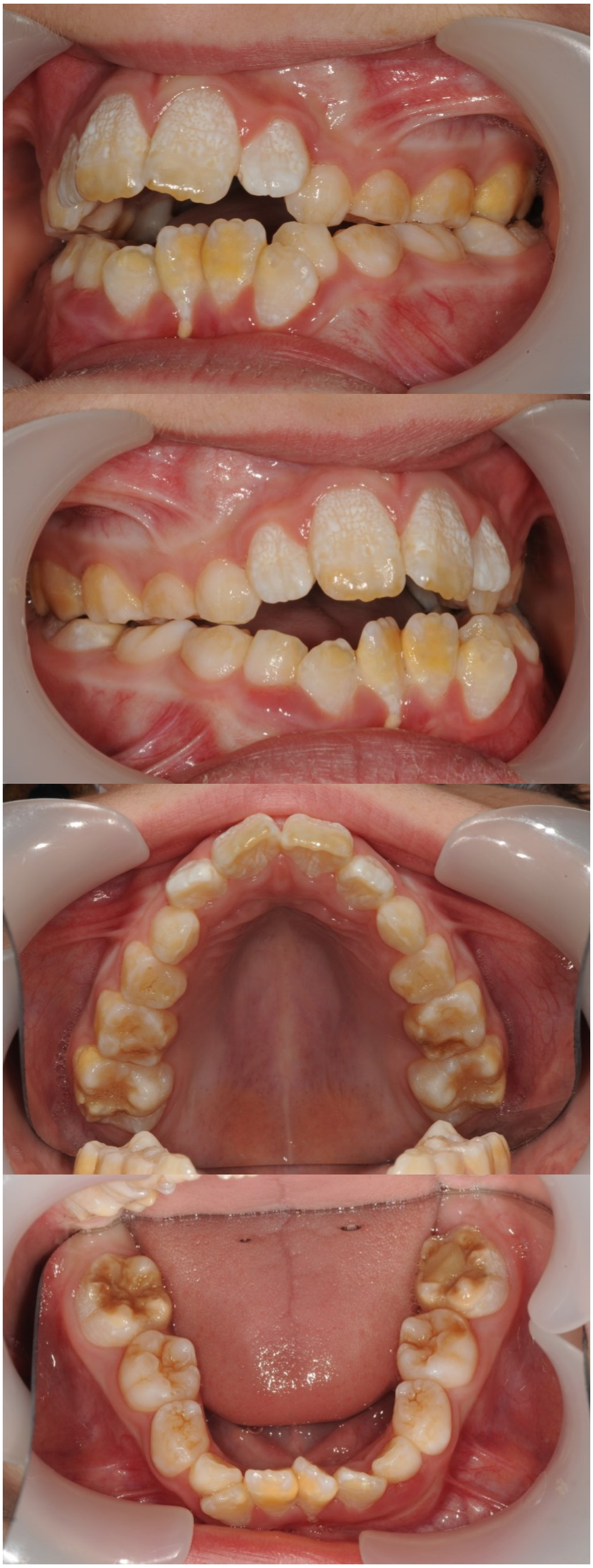

F10 - c.3277+1G>A:p.? - Pathogenic

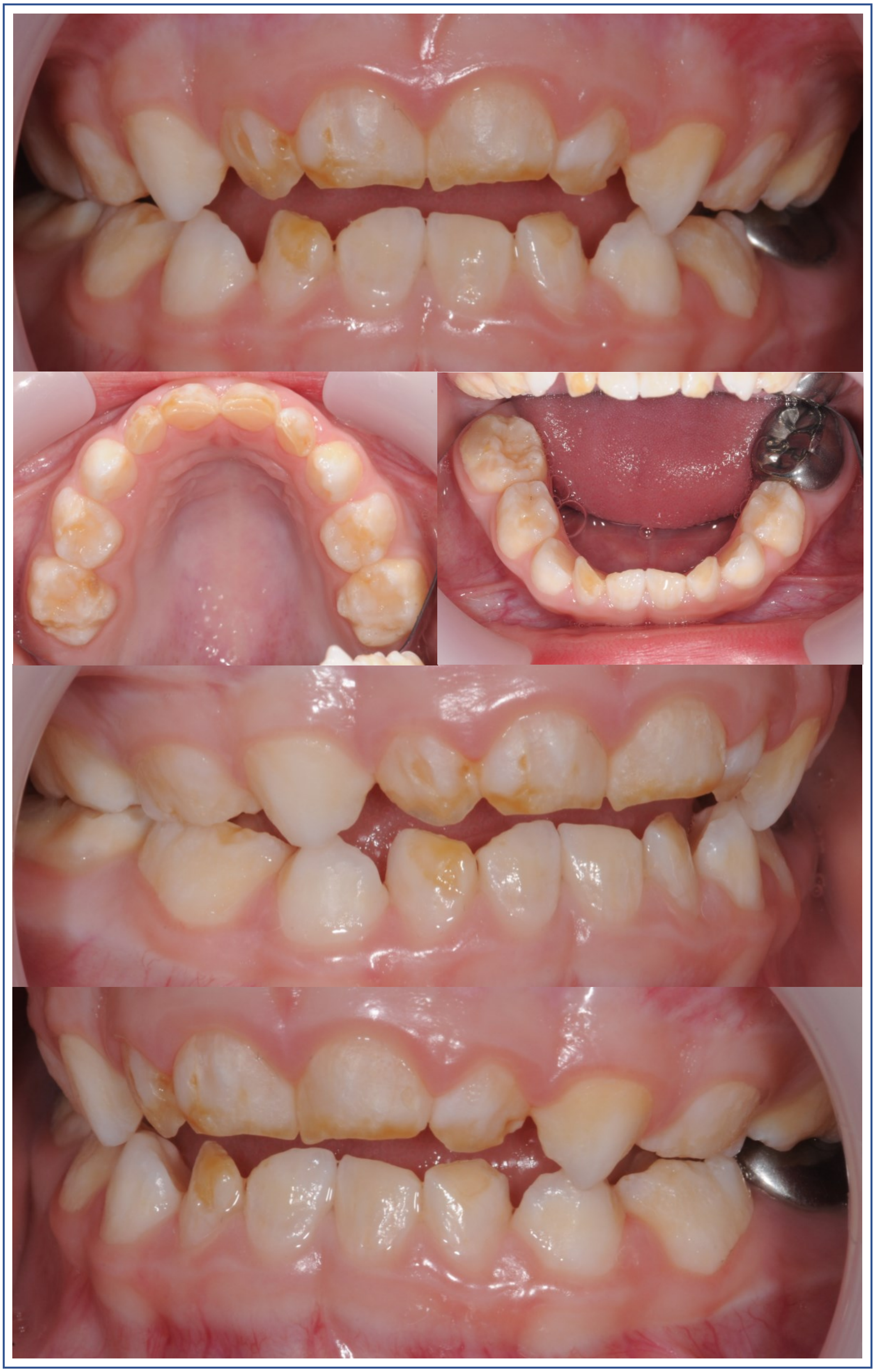

F11 - c.3297C>A:p.(Tyr1099*) - Pathogenic

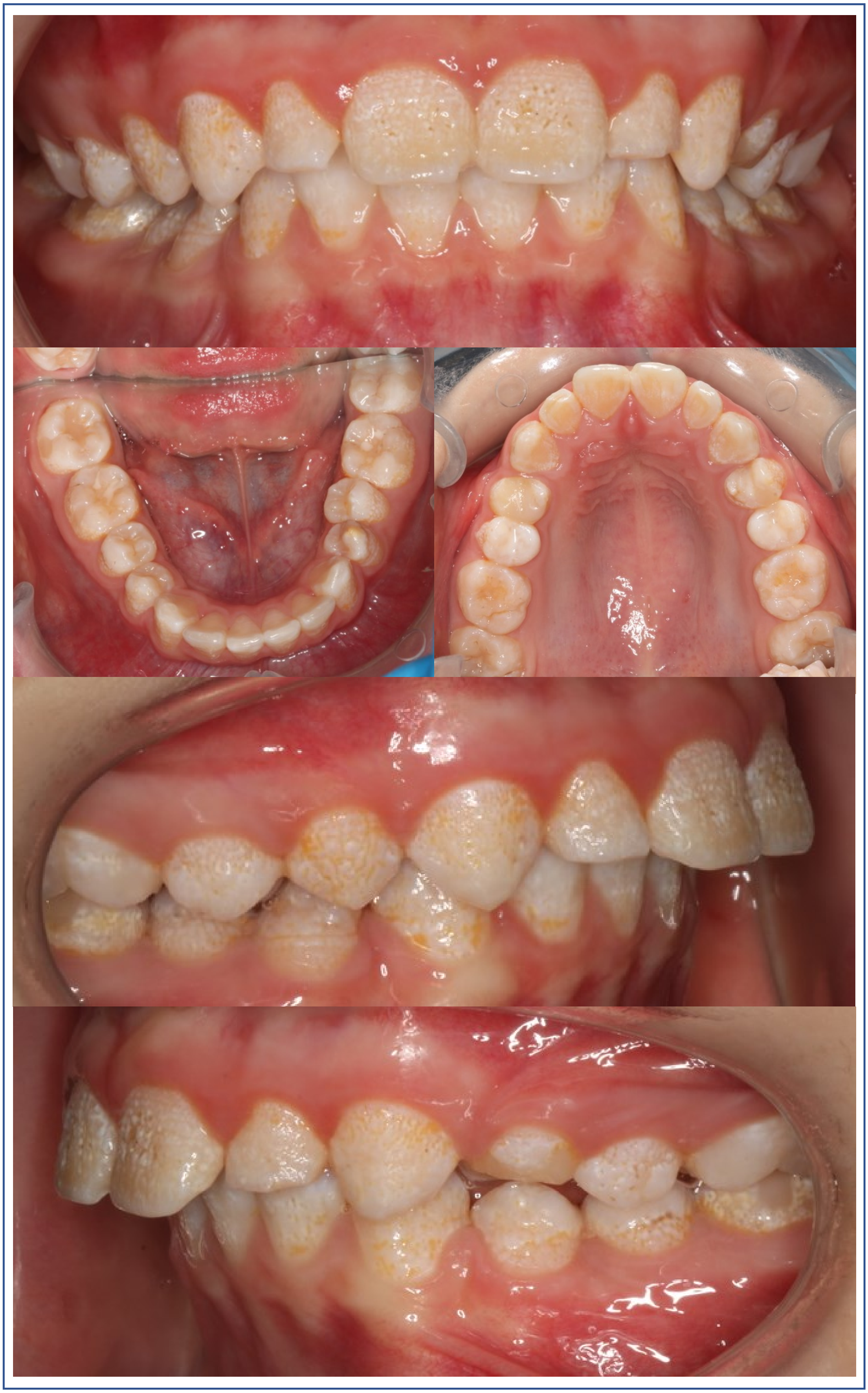

F12 - c.3397C>T:p.(Arg1133Cys) - VUS

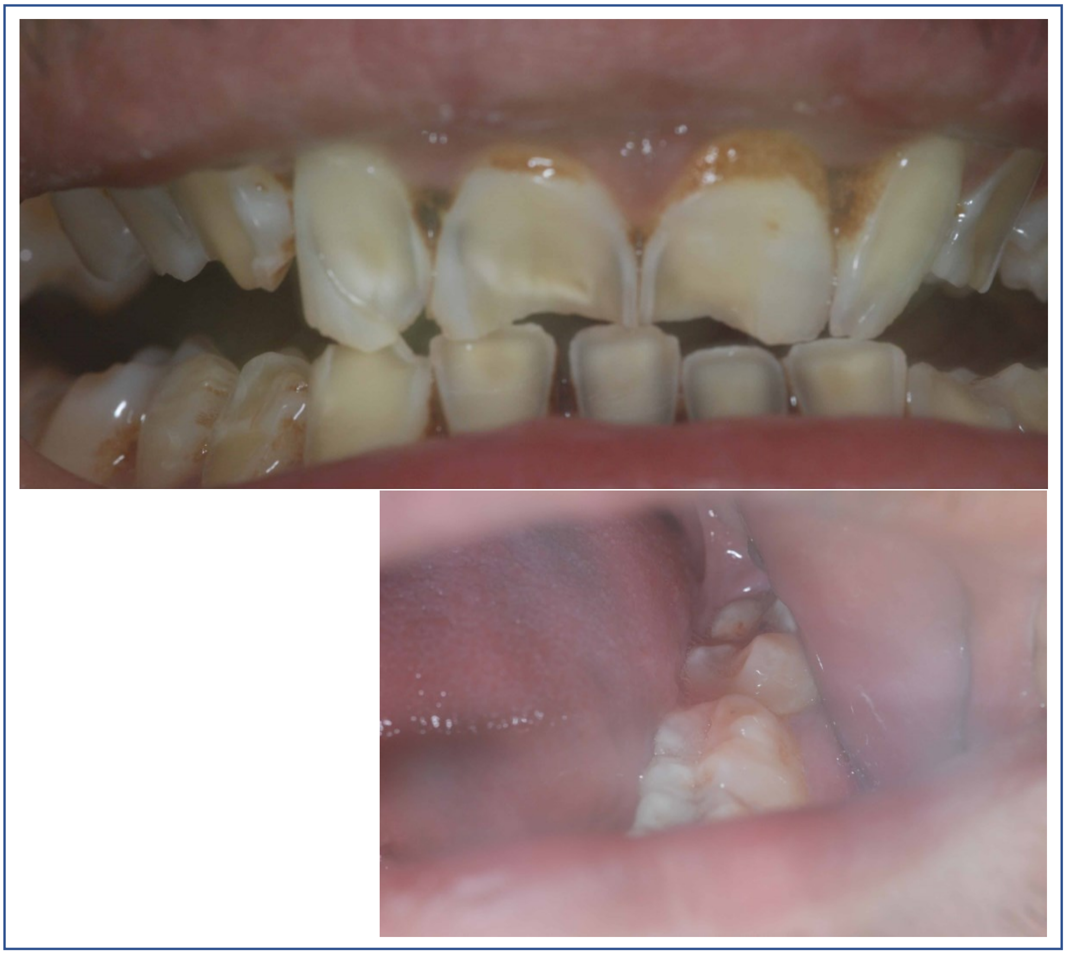

F14 - c.3456del:p.(Pro1154Leufs*97) - Pathogenic

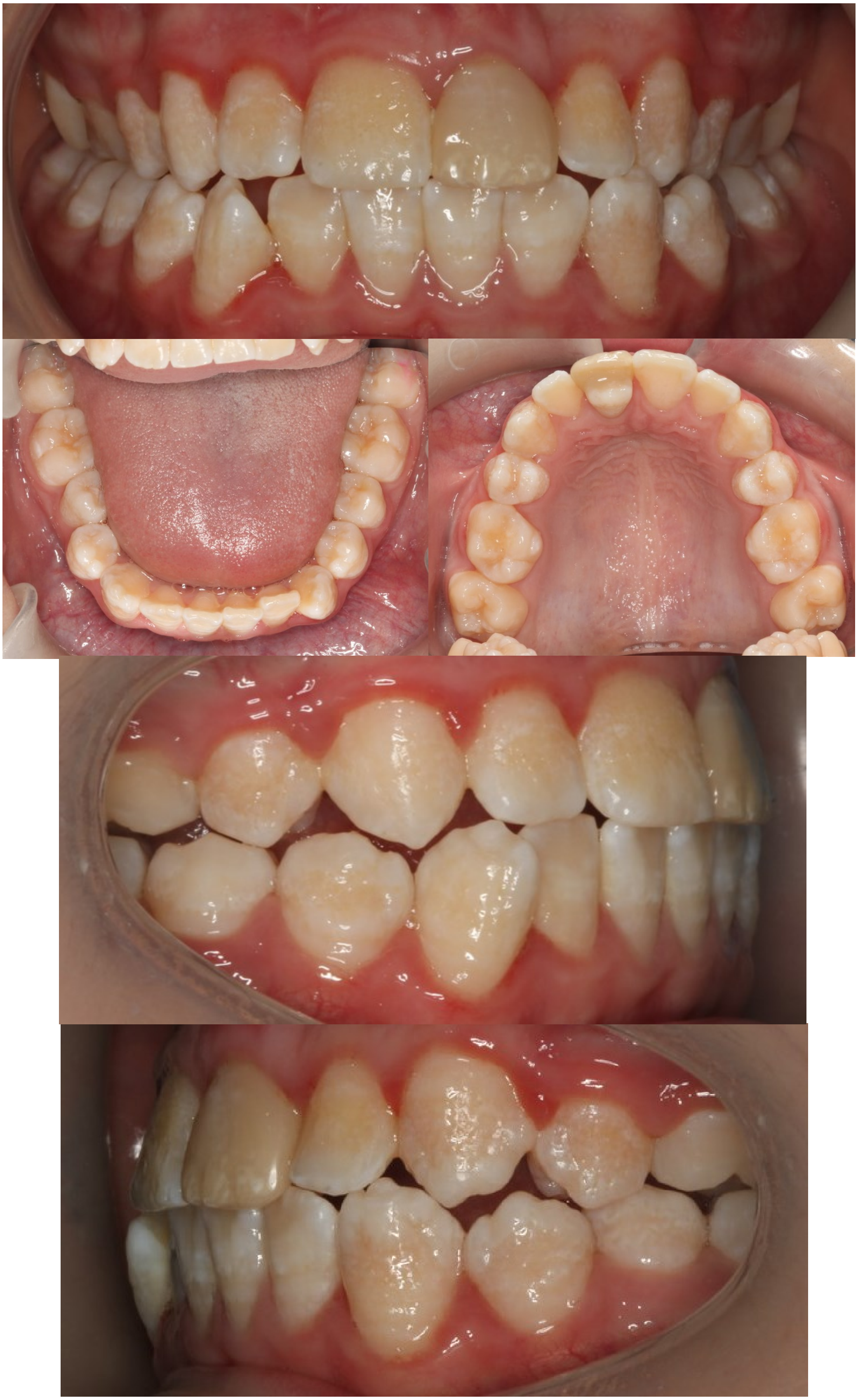

F15 - c.3462_3463del:p.(Gly1155Leufs*7) - Pathogenic

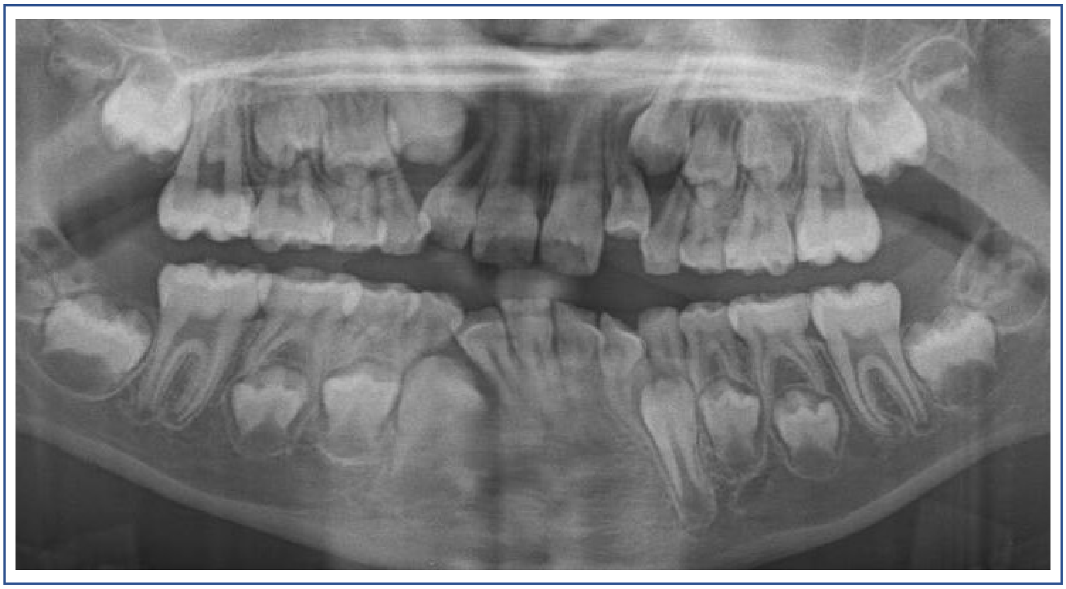

F18 - c.3605C>T:p.(Ser1202Leu) - VUS

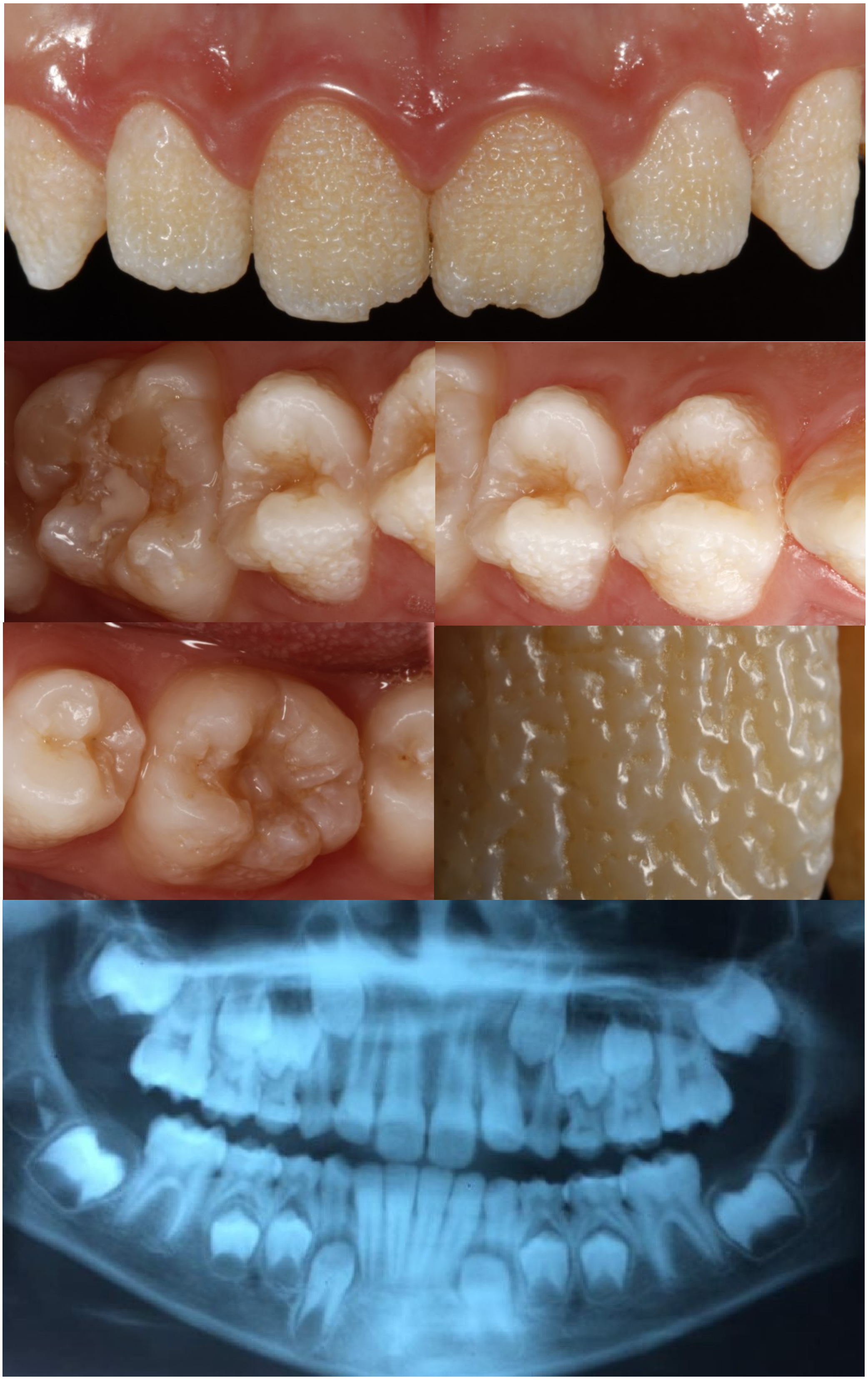

Supplement: Supplementary data [file jmg-2023-109510supp002.pdf]
