# Supplementary material for: Heterozygous COL17A1 variants are a frequent cause of amelogenesis imperfecta
Source: J Med Genet. 2023 Nov 18;61(4):347–55. doi: 10.1136/jmg-2023-109510 (PMC10982616; doi:10.1136/jmg-2023-109510)
Supplement: Supplementary data [file jmg-2023-109510supp001.pdf]

Heterozygous *COL17A1* variants are a frequent cause of Amelogenesis Imperfecta

Ummey Hany[1], Christopher M. Watson[1,2], Lu Liu[1,3], Claire E.L. Smith[1], Asmaa Harfoush[3], James A. Poulter[1], Georgios Nikolopoulos[4], Richard Balmer[3], Catriona J. Brown[5], Anesha Patel[6], Jenny Simmonds[2], Ruth Charlton[2], María Gabriela Acosta de Camargo[7], Helen D Rodd[8], Hussain Jafri[9], Agne Antanaviciute[10], Michelle Moffat[11], Maisoon Al-Jawad[3], Chris F. Inglehearn[1,12], Alan J. Mighell[3,12]

Affiliations

1: Leeds Institute of Medical Research, University of Leeds, St James's University Hospital, Leeds, UK

2: North East and Yorkshire Genomic Laboratory Hub, Central Lab, St. James's University Hospital, Leeds, UK

3: School of Dentistry, Clarendon Way, University of Leeds, Leeds, UK

4: Institute for Fundamental Biomedical Research, B.S.R.C. 'Alexander Fleming', 16672 Vari, Attica, Greece

5: Birmingham Dental Hospital, Mill Pool Way, Edgbaston, Birmingham, UK

6: LCRN West Midlands Core Team, NIHR Clinical Research Network (CRN), Birmingham Research Park (West Wing), Vincent Drive, Edgbaston, Birmingham, UK

7: Department of Dentistry of the Child and Adolescent, Universidad de Carabobo, Venezuela

8: Academic Unit of Oral Health Dentistry and Society, School of Clinical Dentistry, University of Sheffield, Sheffield, S10 2TA, UK

9: Fatima Jinnah Medical University, Lahore, Pakistan

10: MRC Human Immunology Unit, University of Oxford

11: Paediatric Dentistry, The Newcastle Upon Tyne Hospitals NHS Foundation Trust, Newcastle upon Tyne, UK

12: Joint senior authors

Supplementary Files

1. **Table S1:** *COL17A1* variants associated with JEB (Database search) and their distribution in different protein domains.

The *COL17A1* gene encodes a transmembrane protein collagen XVII consisting of 1497 amino acid residues. The amino acid residues in different domains are as defined below [1, 2].

Domains	Exons	Amino Acid residues	Total Amino Acid
Endodomain	exon 2-exon 17	1-466	466
Transmembrane	exon 17	466-489	23
Ectodomain	exon 18-exon 56	490-1498	1008
			1497

By combining a literature search on the NCBI database (https://pubmed.ncbi.nlm.nih.gov/) with access to the HGMD professional database, we identified 232 *COL17A1* variants reported to cause JEB [3]. 172 of these were located in the ectodomain, 3 in the transmembrane domain and 57 in the endodomain.

Variant	Exonic Function	Domain
c.-11-2A>G	Splice	Endodomain
c.2T>A;p.0?	Missense	Endodomain
c.2T>C;p.0?	Missense	Endodomain
c.25C>T;p.(Arg9*)	Premature termination codon	Endodomain
c.51+1G>A	Splice	Endodomain
c.56delT;p.(Val19Alafs*5)	Frameshift small indels	Endodomain
c.82dupA;p.(Thr28Asnfs*15)	Frameshift small indels	Endodomain
c.158del; p.(Glu53fs)	Frameshift small indels	Endodomain
c.202delA;p.(Thr68Leufs*106)	Frameshift small indels	Endodomain
c.209-210insCA	Frameshift small indels	Endodomain
c.213-214dupAC;p.(Arg72Hisfs*103)	Frameshift small indels	Endodomain
c.214C>T:p.(Arg72*)	Premature termination codon	Endodomain
c.366dup;p.(Arg123fs)	Frameshift small indels	Endodomain
c.372_373insA;p.(Glu125Argfs*17)	Frameshift small indels	Endodomain
c.380-1G>A	Splice	Endodomain
c.412C>T;p.(Arg138*)	Premature termination codon	Endodomain
c.418_419delAG;p.(Ser140*)	Frameshift small indels	Endodomain
c.426delT p.(Ile142fs)	Frameshift small indels	Endodomain
c.427C>T;p.(Arg143*)	Premature termination codon	Endodomain
c.433C>T;p.(Arg145*)	Premature termination codon	Endodomain
c.460C>T;p.(Arg154*)	Premature termination codon	Endodomain
c.464-2A>G	Splice	Endodomain
c.505C>T;p.(Arg169*)	Premature termination codon	Endodomain
c.520_521del;p.(Ser174fs)	Frameshift small indels	Endodomain
c.558_567del;p.(Lys187Leufs*7)	Frameshift small indels	Endodomain
c.569-2A>G	Splice	Endodomain

c.621C>G;p.(Tyr207*)	Premature termination codon	Endodomain
c.655dupC;p.(His219Profs*118)	Frameshift small indels	Endodomain
c.675_688dup14;p.(Ser230Cysfs*14)	Frameshift small indels	Endodomain
c.718delA;p.(Thr240Profs*52)	Frameshift small indels	Endodomain
c.772G>A;p.(Gly258Arg)	Missense	Endodomain
c.772G>T;p.(Gly258*)	Premature termination codon	Endodomain
c.779delC;p.(Pro260Glnfs*32)	Frameshift small indels	Endodomain
c.783delC;p.(Asn261Lysfs*31)	Frameshift small indels	Endodomain
c.794C>G;p.(Ser265Cys)	Missense	Endodomain
c.823delC;p.(Ser275fs)	Frameshift small indels	Endodomain
c.884delC;p.(His296fs)	Frameshift small indels	Endodomain
c.980-1G>C	Splice	Endodomain
c.997C>T;p.(Glu333*)	Premature termination codon	Endodomain
c.1139C>T;p.(Ala380Val)	Missense	Endodomain
c.1141+2T>C	Splice	Endodomain
c.1141+5G>A	Splice	Endodomain
c.1179delA;p.(Ala394Leufs*9)	Frameshift small indels	Endodomain
c.1239_1240delGT;p.(Ser414Hisfs*87)	Frameshift small indels	Endodomain
c.1260delC;p.(Thr421Leufs*72)	Frameshift small indels	Endodomain
c.1267+1G>T	Splice	Endodomain
c.1267+2T>C	Splice	Endodomain
c.1268-2A>G	Splice	Endodomain
p.Ile18del389	Large deletion (exon2-15)	Endodomain
c.1268-267_1465+369del834;p.(Asp423Aladel66)	Large Deletion	Endodomain+Transmembrane
c.1284delA;p.(Ser429fs)	Frameshift small indels	Endodomain
c.1285delA;p.(Ser429fs)	Frameshift small indels	Endodomain
c.1336G>A;p.(Gly446Ser)	Missense	Endodomain
c.1365delC;p.(Trp455fs)	Frameshift small indels	Endodomain
c.1372+1G>T	Splice	Endodomain
c.1374C>A;p.(Cys458*)	Premature termination codon	Endodomain
c.1392G>A;p.(Trp464*)	Premature termination codon	Endodomain
c.1395G>A;p.(Trp465*)	Premature termination codon	Endodomain
c.1445T>C;p.(Leu482Pro)	Missense	Transmembrane
c.1465+2T>C	Splice	Transmembrane
c.1480_1482dupAAG;p.(Lys494dup)	In frame small indel	Ectodomain
c.1490_1491delCGinsT;p.(Ala497Valfs*23)	Frameshift small indels	Ectodomain
c.1507delG;p.(Glu503Argfs*17)	Frameshift small indels	Ectodomain
c.1601delA;p.(Asp534Alafs*19)	Frameshift small indels	Ectodomain
c.1601_1602insG;p.(Asp534Glufs*10)	Frameshift small indels	Ectodomain
c.1612delA;p.(Ile538Leufs*15)	Frameshift small indels	Ectodomain
c.1616G>A;p.(Gly539Glu)	Missense	Ectodomain
c.1696C>T;p.(Arg566*)	Premature termination codon	Ectodomain
c.1706delA;p.(Pro569fs)	Frameshift small indels	Ectodomain
c.1745-2A>C	Splice	Ectodomain
c.1745-2A>G	Splice	Ectodomain
c.1750C>T;p.(Arg584*)	Premature termination codon	Ectodomain

c.1772-2A>C	Splice	Ectodomain
c.1817G>A;p.(Gly606Asp)	Missense	Ectodomain
c.1826G>A;p.(Gly609Asp)	Missense	Ectodomain
c.1834G>A;p.(Gly612Arg)	Missense	Ectodomain
c.1834G>C;p.(Gly612Arg)	Missense	Ectodomain
c.1852G>A;p.(Gly618Ser)	Missense	Ectodomain
c.1861G>A;p.(Gly621Ser)	Missense	Ectodomain
c.1877-2A>C	Splice	Ectodomain
c.1880G>T;p.(Gly627Val)	Missense	Ectodomain
c.1880delG;p.(Gly627Alafs*56)	Frameshift small indels	Ectodomain
c.1898G>A;p.(Gly633Asp)	Missense	Ectodomain
c.1992_1995delGGGT;p.(Gly665Profs*17)	Frameshift small indels	Ectodomain
c.2002+2T>G	Splice	Ectodomain
c.2003-1G>C	Splice	Ectodomain
c.2062delC;p.(Arg688Glufs*4)	Frameshift small indels	Ectodomain
c.2062C>T;p.(Arg688*)	Premature termination codon	Ectodomain
c.2227+153_2336-318del	Large Deletion	Ectodomain
c.2228-101_2263+70delins15	Frameshift small indels	Ectodomain
c.2237delG;p.(Gly746Alafs*53)	Frameshift small indels	Ectodomain
c.2240delC;p.(Pro747Glnfs*52)	Frameshift small indels	Ectodomain
c.2251C>T;p.(Gln751*)	Premature termination codon	Ectodomain
c.2282_2283delGG;p.(Gly761Aspfs*40)	Frameshift small indels	Ectodomain
c.2336-1G>T	Splice	Ectodomain
c.2336-2A>G	Splice	Ectodomain
c.2342delG;p.(Thr781fs)	Frameshift small indels	Ectodomain
c.2350C>T;p.(Gln784*)	Premature termination codon	Ectodomain
c.2363-2A>G	Splice	Ectodomain
c.2363dup;p.(Leu789Thrfs*13)	Frameshift small indels	Ectodomain
c.2383C>T;p.(Arg795*42)	Premature termination codon	Ectodomain
c.2407G>T;p.(Gly803*)	Premature termination codon	Ectodomain
c.2434+1G>A	Splice	Ectodomain
c.2441-2A>G	Splice	Ectodomain
c.2441-1G>T	Splice	Ectodomain
c.2468-2A>G	Splice	Ectodomain
c.2468C>A;p.(Pro823Gln)	Missense	Ectodomain
c.2488G>A;p.(Gly830Arg)	Missense	Ectodomain
c.2496dupT;p.(Gly833Argfs*22)	Frameshift small indels	Ectodomain
c.2518del10	Frameshift small indels	Ectodomain
c.2520dupT;p.(Ala841Cysfs*14)	Frameshift small indels	Ectodomain
c.2544delA;p.(His849Ilefs*217)	Frameshift small indels	Ectodomain
c.2551+1G>A	Splice	Ectodomain
c.2561_2565delATTTA;p.(Asn854Thrfs*109)	Frameshift small indels	Ectodomain
c.2563_2564delTT;p.(Leu855Thrfs*109)	Frameshift small indels	Ectodomain
c.2564T>G;p.(Leu855*)	Premature termination codon	Ectodomain
c.2564T>A;p.(Leu855*)	Premature termination codon	Ectodomain
c.2566C>T;p.(Gln856*)	Premature termination codon	Ectodomain

c.2585_2586delCC;p.(Pro862Argfs*102)	Frameshift small indels	Ectodomain
c.2635C>T;p.(Arg879*)	Premature termination codon	Ectodomain
c.2666delTT	Frameshift small indels	Ectodomain
c.2690insT;p.(Ser898fs)	Frameshift small indels	Ectodomain
c.2706dup;p.(Phe903Leufs*62)	Frameshift small indels	Ectodomain
c.2716G>A;p.(Gly906Ser)	Missense	Ectodomain
c.2723dupC;p.(Gly909Argfs*56)	Frameshift small indels	Ectodomain
c.2725G>C;p.(Gly909Arg)	Missense	Ectodomain
c.2776delA;p.(Arg926Glufs*140)	Frameshift small indels	Ectodomain
c.2840_2844delTCAAC;p.(Leu947Profs*16)	Frameshift small indels	Ectodomain
c.2861delG;p.(Gly954Alafs*112)	Frameshift small indels	Ectodomain
c.2875delC;p.(Gln959Argfs*107)	Frameshift small indels	Ectodomain
c.2881delA	Frameshift small indels	Ectodomain
c.2897-9G>A	Splice	Ectodomain
c.2897-2A>C	Splice	Ectodomain
c.2944del;p.(Glu982Lysfs84*)	Frameshift small indels	Ectodomain
c.2948-1G>C	Splice	Ectodomain
c.2965delG;p.(Met989fs)	Frameshift small indels	Ectodomain
c.2971G>A;p.(Val991Met)	Missense	Ectodomain
c.2972delT;p.(Val991Glyfs*75)	Frameshift small indels	Ectodomain
c.2975C>A;p.(Ser992*)	Premature termination codon	Ectodomain
c.2993dupC;p.(Gly999Trpfs*22)	Frameshift small indels	Ectodomain
c.3000-3008del;p.(Pro1003_Gly1005del)	In frame small indel	Ectodomain
c.3002-2A>C	Splice	Ectodomain
c.3046C>T;p.(Gln1016*)	Premature termination codon	Ectodomain
c.3053-1G>C	Splice	Ectodomain
c.3067C>T;p.(Gln1023*)	Premature termination codon	Ectodomain
c.3071-6C>A	Splice	Ectodomain
c.3131delC;p.(Pro1044Glnfs*22)	Frameshift small indels	Ectodomain
c.3164delT;p.(Phe1055Serfs*11)	Frameshift small indels	Ectodomain
c.3171_3173delCTC;p.(Tyr1057*)	Frameshift small indels	Ectodomain
c.3175delG;p.(Glu1059Serfs*7)	Frameshift small indels	Ectodomain
c.3193_3208del16;p.(Val1065Leufs*35)	Frameshift small indels	Ectodomain
c.3198C>T;p.(Ser1066Ser	Splice	Ectodomain
c.3205C>T;p.(Arg1069Trp)	Missense	Ectodomain
c.3236delC;p.(Ser1079Cysfs*26)	Frameshift small indels	Ectodomain
c.3269dupT;p.(Leu1091Alafs*5)	Frameshift small indels	Ectodomain
c.3275A>C;(Gln1092Pro)	Missense	Ectodomain
c.3288_3295del8;p.(Arg1097Profs*33)	Frameshift small indels	Ectodomain
c.3292C>T;p.(Gln1098*)	Premature termination codon	Ectodomain
c.3301C>T;p.(Arg1101Cys)	Missense	Ectodomain
c.3327delT;p.(Pro1110Argfs*21)	Frameshift small indels	Ectodomain
c.3408delC;p.(Tyr1137Thrfs*114)	Frameshift small indels	Ectodomain
c.3481dupT;p.(Tyr1161fs*2)	Frameshift small indels	Ectodomain
c.3482_3483del;p.(Tyr1161*)	Frameshift small indels	Ectodomain
c.3487G>T;p.(Glu1163*)	Premature termination codon	Ectodomain

c.3496_3497delTC;p.(Ser1166Leufs*6)	Frameshift small indels	Ectodomain
c.3505C>T;p.(Arg1169*)	Premature termination codon	Ectodomain
c.3509-1G>C	Splice	Ectodomain
c.3513delC;p.(Glu1172fs)	Frameshift small indels	Ectodomain
c.3514ins25	Frameshift small indels	Ectodomain
c.3539dupC;p.(Pro1180Profs*62)	Frameshift small indels	Ectodomain
c.3548delC;p.(Pro1183Argfs*68)	Frameshift small indels	Ectodomain
c.3569dupG;p.(Asn1191Glnfs*51)	Frameshift small indels	Ectodomain
c.3569_3570insT;p.(Asn1191Glnfs*51)	Frameshift small indels	Ectodomain
c.3579G>A;p.(Trp1193*)	Premature termination codon	Ectodomain
c.3600-3601delCT	Frameshift small indels	Ectodomain
c.3613_3616delTTAC;p.(Leu1205Ilefs*45)	Frameshift small indels	Ectodomain
c.3615_3619dupACATA	Frameshift small indels	Ectodomain
c.3619+2T>C	Splice	Ectodomain
c.3673_3674insT;p.(Pro1225fs)	Frameshift small indels	Ectodomain
c.3676C>T;p.(Arg1226*)	Premature termination codon	Ectodomain
c.3686C>T;p.(Pro1229Leu)	Missense	Ectodomain
c.3689dup;p.(V1231Cfs*11)	Frameshift small indels	Ectodomain
c.3730G>A;p.(Asp1244Asn)	Missense	Ectodomain
c.3740G>A;p.(Arg1247Gln)	Missense	Ectodomain
c.3766+1G>A	Splice	Ectodomain
c.3766+1G>C	Splice	Ectodomain
c.3782G>C;p.(Ser1261Thr)	Missense	Ectodomain
c.3786_3789delCATT;p.(Phe1262Leufs*49)	Frameshift small indels	Ectodomain
c.3801insC;p.(Gly1268Argfs*25)	Frameshift small indels	Ectodomain
c.3806delC;p.(Pro1269Leufs*43)	Frameshift small indels	Ectodomain
c.3821_3829delGACCCCCTGinsC;p.(Gly1274Alafs*16)	Frameshift small indels	Ectodomain
c.3827dupC;p.(Gly1277Trpfs*16)	Frameshift small indels	Ectodomain
c.3865dupA;p.(Ser1289Lysfs*4)	Frameshift small indels	Ectodomain
c.3871+1G>A	Splice	Ectodomain
c.3871+1G>C	Splice	Ectodomain
c.3897_3900delATCT;p.(Val1301Glyfs*10)	Frameshift small indels	Ectodomain
c.3899_3900delCT;p.(Ser1300Cysfs*29)	Frameshift small indels	Ectodomain
c.3908G>A;p.(Arg1303Gln)	Missense	Ectodomain
c.3922delA;p.(Ser1308Alafs*4)	Frameshift small indels	Ectodomain
c.4003delTC	Frameshift small indels	Ectodomain
c.4011T>A;p.(Tyr1337*)	Premature termination codon	Ectodomain
c.4041T>G;p.(Tyr1347*)	Premature termination codon	Ectodomain
c.4045dupG;p.(Ala1349Glyfs*18)	Frameshift small indels	Ectodomain
c.4080insGG;p.(Gly1361fs)	Frameshift small indels	Ectodomain
c.4088delT;p.(Leu1363fs)	Frameshift small indels	Ectodomain
c.4100_4101delTT;p.(Phe1367Cysfs*8)	Frameshift small indels	Ectodomain
c.4144del4;p.(Glu1382fs)	Frameshift small indels	Ectodomain
c.4145_4148delAGAG;p.(Glu1382Alafs*40)	Frameshift small indels	Ectodomain
c.4149_4150insG;p.(Met1384fs)	Frameshift small indels	Ectodomain
c.4153C>T;p.(Gln1385*)	Premature termination codon	Ectodomain

c.4156+1G>C	Splice	Ectodomain
c.4156+1G>A	Splice	Ectodomain
c.4159C>T;p.(Gln1387*)	Premature termination codon	Ectodomain
c.4207C>T;p.(Gln1403*)	Premature termination codon	Ectodomain
c.4230delC;p.(Gly1411Alafs*12)	Frameshift small indels	Ectodomain
c.4231G>A;p.(Gly1411Ser)	Missense	Ectodomain
c.4237_4238insC;p.(Ser1413Thrfs*42)	Frameshift small indels	Ectodomain
c.4261+1G>C	Splice	Ectodomain
c.4265_4266insTT;p.(Thr1423*)	Frameshift small indels	Ectodomain
c.4295-1G>C	Splice	Ectodomain
c.4307_4310dupTTCA;p.(Gln1437Hisfs*19)	Frameshift small indels	Ectodomain
c.4319dupC;p.(Gly1441Trpfs*14)	Frameshift small indels	Ectodomain
c.4320delT;p.(Gln1442Lysfs*70)	Frameshift small indels	Ectodomain
c.4321delT;p.(Gln1442fs)	Frameshift small indels	Ectodomain
c.4324C>T;p.(Gln1442*)	Premature termination codon	Ectodomain
c.4335delC;p.(Met1446fs)	Frameshift small indels	Ectodomain
c.4410-4413dupCATT	Frameshift small indels	Ectodomain
c.4424-5insC	Splice	Ectodomain
c.4425_4426insC;p.(Lys1476fs)	Frameshift small indels	Ectodomain
c.4425delT	Frameshift small indels	Ectodomain
c.4460G>A;p.(Arg1487Gln)	Missense	Ectodomain
c.4463-1G>A	Splice	Ectodomain

Table S2: *COL17A1* variants associated with AI (This study and from database search).

Variant	Exonic Function	Domain	References
c.340;p.(Ser114Valfs*60)	Frameshift small indels	Endodomain	Leeds AI group
c.460C>T;p.(Arg154*)	Premature termination codon	Endodomain	Leeds AI group
c.541_550del;p.(Asn181Profs*13)	Frameshift small indels	Endodomain	Leeds AI group
c.1141+1G>A	Splice	Endodomain	Prasad, M. K., et al. (2016)
c.1646G>A;p.(Trp549*)	Premature termination codon	Ectodomain	Prasad, M. K., et al. (2016)
c.1745-2A>C	Splice	Ectodomain	Bloch-Zupan, A., et al. (2023)
c.1861G>A; p.(Gly621Ser)	Missense	Ectodomain	Leeds AI group
c.1873C>T;p.(Arg625*)	Premature termination codon	Ectodomain	Prasad, M. K., et al. (2016)
c.2011G>A;p.(Gly671Ser)	Missense	Ectodomain	Leeds AI group
c.2030G>A;p.(Gly677Asp)	Missense	Ectodomain	Leeds AI group
c.2407G>T;p.(Gly803*)	Premature termination codon	Ectodomain	Prasad, M. K., et al. (2016)
c.2435-1G>A	Splice	Ectodomain	Leeds AI group
c.2912del;p.(Pro971Glnfs*95)	Frameshift small indels	Ectodomain	Leeds AI group
c.2947+2T>C	Splice	Ectodomain	Leeds AI group
c.3277+1G>A	Splice	Ectodomain	Leeds AI group
c.3297C>A;p.(Tyr1099*)	Premature termination codon	Ectodomain	Leeds AI group
c.3327del;p.(Pro1110Argfs*21)	Frameshift small indels	Ectodomain	Bloch-Zupan, A., et al. (2023)
c.3397C>T;p.(Arg1133Cys)	Missense	Ectodomain	Leeds AI group
c.3456del;p.(Pro1154Leufs*97)	Frameshift small indels	Ectodomain	Leeds AI group
c.3462_3463del;p.(Gly1155fs*7)	Frameshift small indels	Ectodomain	Leeds AI group
c.3595G>C;p.(Glu1199Gln)	Missense	Ectodomain	Leeds AI group
c.3605C>T;p.(Ser1202Leu)	Missense	Ectodomain	Leeds AI group
c.4147_4148del;p.(Ser1383Hisfs*71)	Frameshift small indels	Ectodomain	Leeds AI group

Table S3: *COL17A1* variants associated with ERED (From database search).

Variant	Exonic Function	Domain
c.2816C>T;(Thr939Ile)	Missense	Ectodomain
c.3156C>T	Splice	Ectodomain
c.3554C>T;(Pro1185Leu)	Missense	Ectodomain

Table S4: Genes included in the smMIP reagent. Reference genome GRCh37/hg19.

Gene Name	Gene Symbol	OMIM	Genomic Coordinates	Cytoband
LAMININ, BETA-3	*LAMB3*	150310	chr1:209,788,218-209,825,820	1q32.2
INTEGRIN, BETA-6	*ITGB6*	147558	chr2:160,958,233-161,056,589	2q24.2
AMELOTIN	*AMTN*	610912	chr4:71,384,298-71,398,459	4q13.3
AMELOBLASTIN	*AMBN*	601259	chr4:71,457,975-71,473,004	4q13.3
ENAMELIN	*ENAM*	606585	chr4:71,494,461-71,512,536	4q13.3
ODONTOGENESIS-ASSOCIATED PHOSPHOPROTEIN	*ODAPH*	614829	chr4:76,481,258-76,491,103	4q21.1
FAMILY WITH SEQUENCE SIMILARITY 83	*FAM83H*	611927	chr8:144,806,103-144,815,914	8q24.3
COLLAGEN, TYPE XVII, ALPHA-1	*COL17A1*	113811	chr10:105,791,046-105,845,638	10q25.1
RECEPTOR EXPRESSED IN LYMPHOID TISSUES	*RELT*	611211	chr11:73,087,405-73,108,519	11q13.4
MATRIX METALLOPROTEINASE 20	*MMP20*	604629	chr11:102,447,566-102,496,063	11q22.2
G PROTEIN-COUPLED RECEPTOR 68	*GPR68*	601404	chr14:91,698,876-91,710,852	14q32.11
SOLUTE CARRIER FAMILY 24 (SODIUM/POTASSIUM/CALCIUM EXCHANGER), MEMBER 4	*SLC24A4*	609840	chr14:92,790,152-92,967,825	14q32.12
WD REPEAT-CONTAINING PROTEIN 72	*WDR72*	613214	chr15:53,805,938-54,051,859	15q21.3
TRANSCRIPTION FACTOR Sp6	*SP6*	608613	chr17:45,922,280-45,928,516	17q21.32
DISTAL-LESS HOMEOBOX 3	*DLX3*	600525	chr17:48,067,369-48,072,588	17q21.33
FAMILY WITH SEQUENCE SIMILARITY 20, MEMBER A	*FAM20A*	611062	chr17:66,531,257-66,597,095	17q24.2
ACID PHOSPHATASE 4	*ACP4*	606362	chr19:51,293,672-51,298,481	19q13.33
KALLIKREIN-RELATED PEPTIDASE 4	*KLK4*	603767	chr19:51,409,608-51,413,994	19q13.41
AMELOGENIN	*AMELX*	300391	chrX:11,311,533-11,318,881	Xp22.2

Figure S1: A comparison of *COL17A1* variants detected in AI and JEB.

A. Collagen XVII variant types reported in AI (outer ring) and JEB (inner ring) patients: Most variants detected in both AI and JEB are those that lead to frameshift and PTC. Fewer variants are reported in AI (n=23) than in JEB (n=232). The differences observed were not significant.

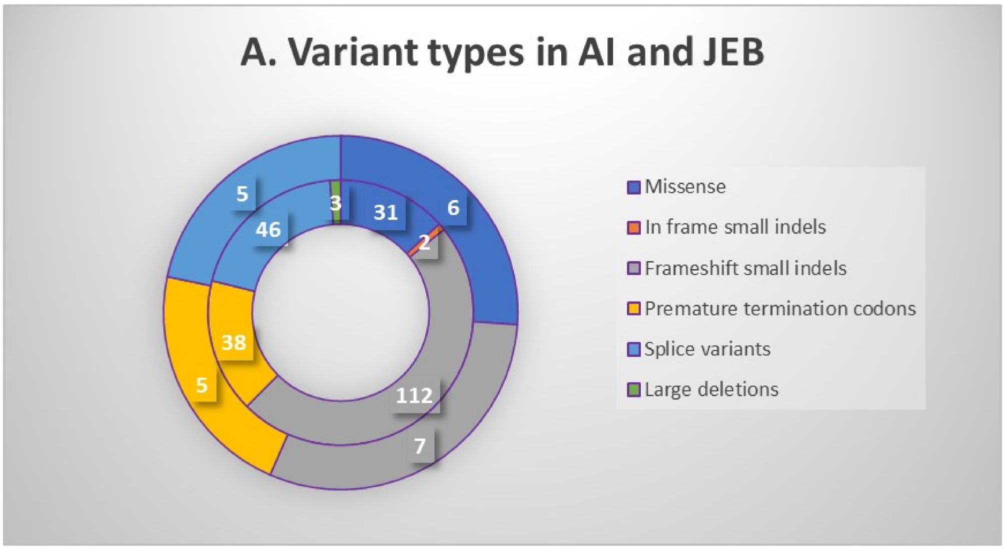

B. Variant distribution in different domains of collagen XVII reported in AI (outer ring) and JEB (inner ring) patients: A total of 23 variants are reported to be associated with AI, 19 of them occurring in the ectodomain and 4 of them in endodomain. A total of 232 variants were reported to be associated with JEB, 172 of them were occurring in ectodomain, 57 in endodomain and 3 of them in transmembrane domain.

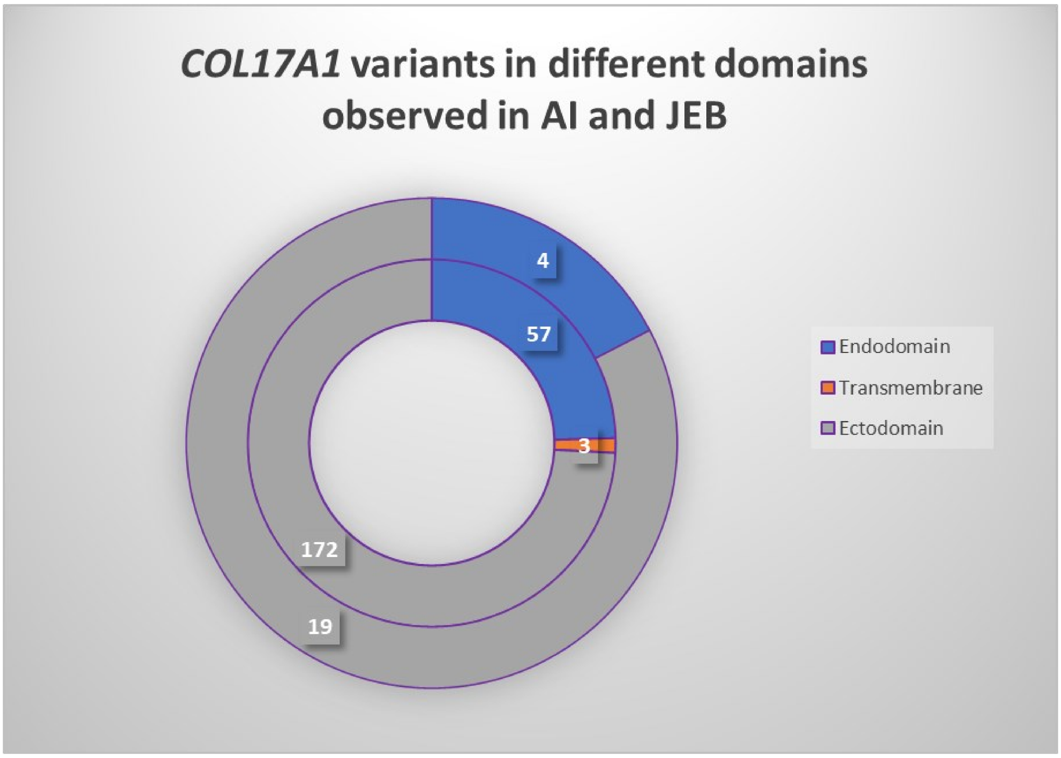

Figure S2: Family pedigree and IGV trace of the variant c.340; p.(Ser114Valfs*60) detected in the family 19, generated by using human reference genome hg19.

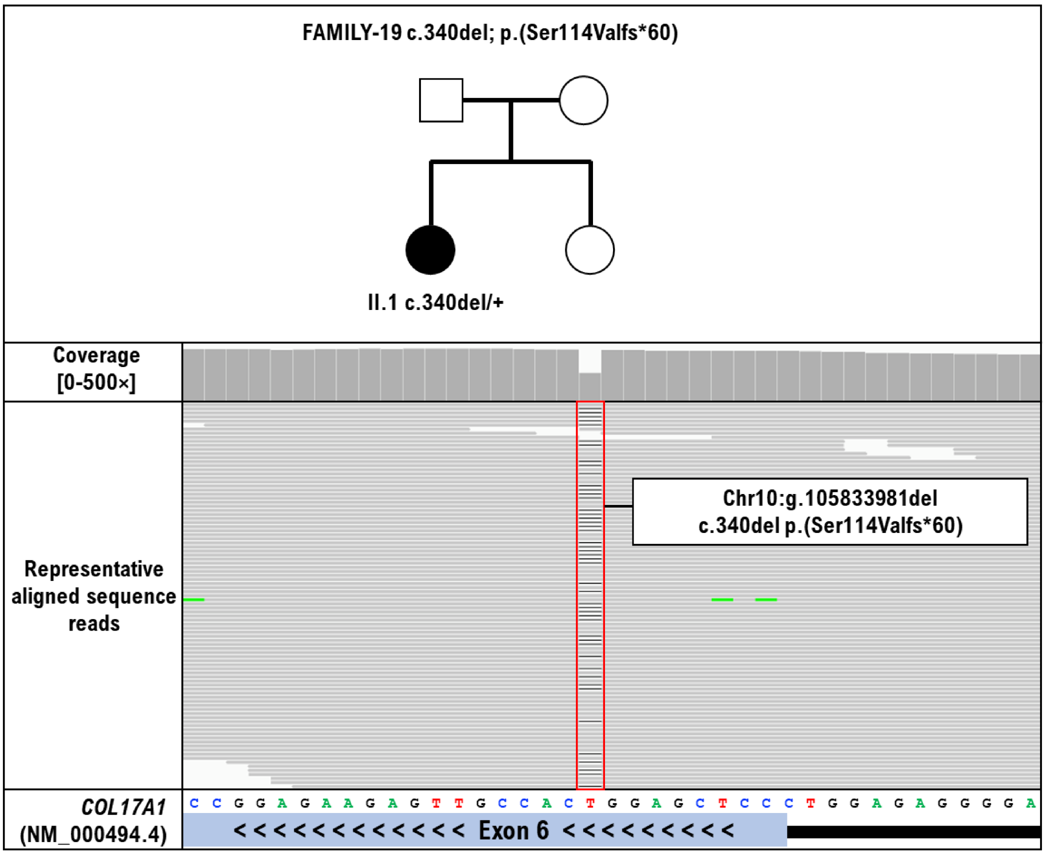

Figure S3: Surface pitting observed in primary teeth from different families.
Whole tooth images from the proband of (i) F14, (ii and iii) F9. SEM cross-section images showing surface pit (iv) F9 (inset), (v) Higher resolution of the pit.

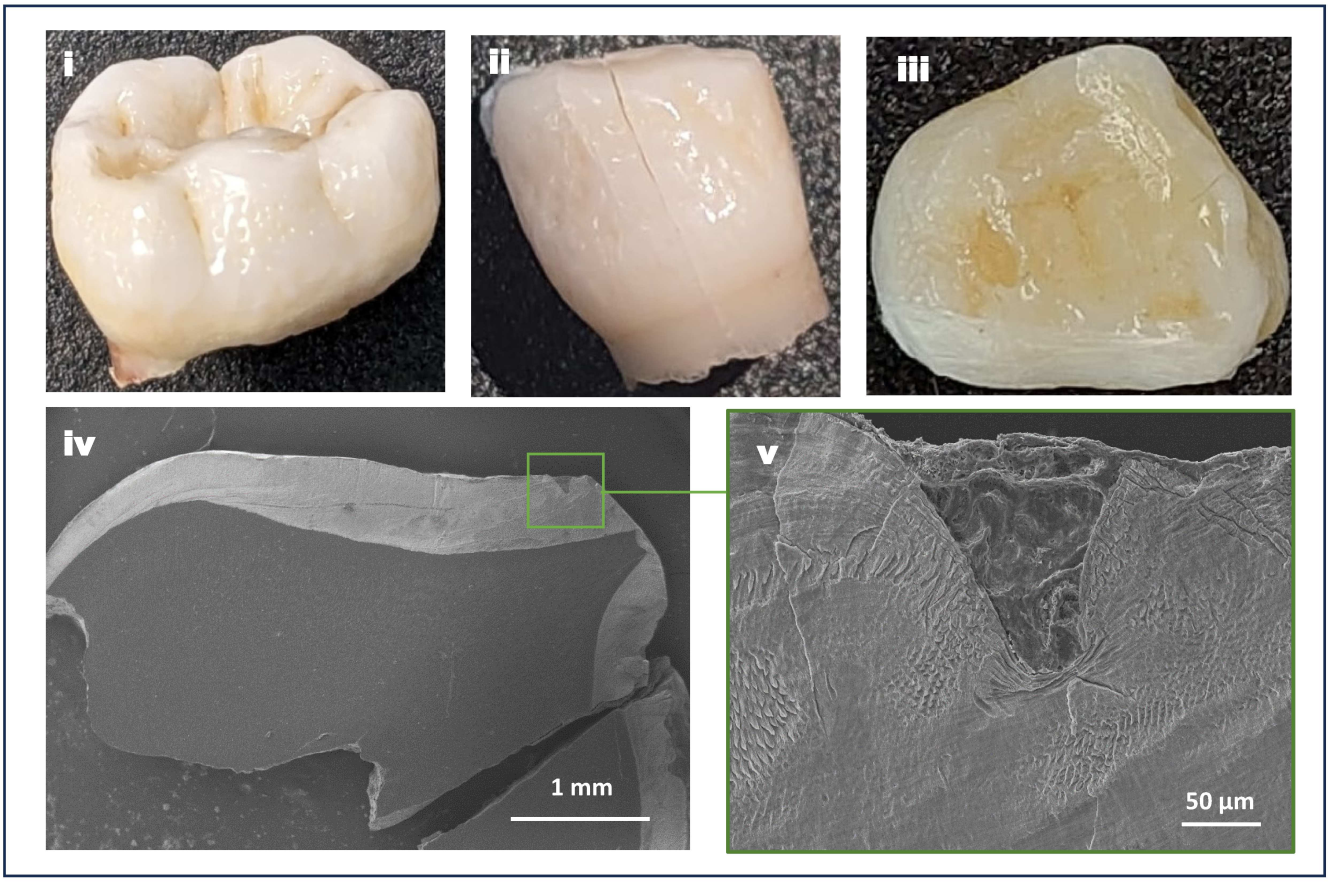

References

1. Giudice, G.J., D.J. Emery, and L.A. Diaz, *Cloning and primary structural analysis of the bullous pemphigoid autoantigen BP180.* Journal of Investigative Dermatology, 1992. **99**(3): p. 243-250.
2. Areida, S.K., et al., *Properties of the Collagen Type XVII Ectodomain: EVIDENCE FOR N- TO C-TERMINAL TRIPLE HELIX FOLDING *.* Journal of Biological Chemistry, 2001. **276**(2): p. 1594-1601.
3. Stenson, P.D., et al., *The Human Gene Mutation Database: towards a comprehensive repository of inherited mutation data for medical research, genetic diagnosis and next-generation sequencing studies.* Hum Genet, 2017. **136**(6): p. 665-677.
